# Application of Pharmacokinetic/Pharmacodynamic Modeling to Bridge Mouse Antitumor Efficacy and Monkey Toxicology Data for Determining the Therapeutic Index of an Interleukin-10 Fc Fusion Protein

**DOI:** 10.3389/fphar.2022.829063

**Published:** 2022-06-20

**Authors:** Zheng Yang, James Loy, Brian Poirson, Yanshan Dai, Surendran Rajendran, Shihua Xu, Vanessa Spires, Murali Gururajan, Zheng Lin, Jaren Arbanas, Stephen Carl, Samantha Pace, Yun Wang, John Mehl, Krishna Vasudevan, Thomas Spires, Ruslan Novosiadly, Shodeinde Coker, Raymond Perez, Kelly Covello, Paul Morin, Robert Graziano, Miranda Broz, Lois Lehman-McKeeman

**Affiliations:** ^1^ Metabolism and Pharmacokinetics, Pharmaceutical Candidate Optimization, Bristol Myers Squibb, Princeton, NJ, United States; ^2^ Discovery Toxicology, Pharmaceutical Candidate Optimization, Bristol Myers Squibb, Cambridge, MA, United States; ^3^ Discovery Oncology, Bristol Myers Squibb, Princeton, NJ, United States; ^4^ Nonclinical Disposition and Bioanalysis, Bristol Myers Squibb, Princeton, NJ, United States; ^5^ Discovery Biotherapeutics, Bristol Myers Squibb, Princeton, NJ, United States; ^6^ Discovery Pharmaceutics and Analytical Sciences, Pharmaceutical Candidate Optimization, Bristol Myers Squibb, Princeton, NJ, United States; ^7^ Translational Medicine, Bristol Myers Squibb, Princeton, NJ, United States; ^8^ Oncology Early Clinical Development, Bristol Myers Squibb, Princeton, NJ, United States; ^9^ Discovery Oncology, Bristol Myers Squibb, Redwood City, CA, United States; ^10^ Pharmaceutical Candidate Optimization, Bristol Myers Squibb, Princeton, NJ, United States

**Keywords:** pharmacokinetic/pharmacodynamic modeling, Fc-fusion protein, IL-10, IL-18, therapeutic index, pharmacodynamic biomarker, anemia (therapy), thrombocytopenia (therapy)

## Abstract

Pharmacokinetic/pharmacodynamic (PK/PD) modeling was performed to quantitatively integrate preclinical pharmacology and toxicology data for determining the therapeutic index (TI) of an interleukin-10 (IL-10) fragment crystallizable (Fc) fusion protein. Mouse Fc fused with mouse IL-10 (mFc-mIL-10) was studied in mice for antitumor efficacy, and the elevation of interleukin-18 (IL-18) was examined as a PD biomarker. The *in vivo* mFc-mIL-10 EC_50_ for the IL-18 induction was estimated to be 2.4 nM, similar to the *in vitro* receptor binding affinity (K_d_) of 3.2 nM. The IL-18 induction was further evaluated in cynomolgus monkeys, where the *in vivo* induction EC_50_ by a human IL-10 human Fc-fusion protein (hFc-hIL-10) was 0.08 nM vs. 0.3 nM measured as the *in vitro* K_d_. The extent of the IL-18 induction correlated with mouse antitumor efficacy and was used to connect mouse efficacy to that in monkeys. The PD-based efficacious dose projected in monkeys was comparable to the results obtained using a PK-based method in which mouse efficacious exposure was targeted and corrected for affinity differences between the species. Furthermore, PK/PD relationships were developed for anemia and thrombocytopenia in monkeys treated with hFc-hIL-10, with thrombocytopenia predicted to be dose-limiting toxicity. Using quantitative pharmacology and toxicology information obtained through modeling work in the same species, the TI of hFc-hIL-10 in monkeys was determined to be 2.4 (vs. PD-based efficacy) and 1.2–3 (vs. PK-based efficacy), indicating a narrow safety margin. The model-based approaches were proven valuable to the developability assessment of the IL-10 Fc-fusion protein.

## 1 Introduction

The therapeutic index (TI) or safety margin is classically defined as the dose ratio between the dose causing toxicity and the dose producing desired pharmacological effects (Muller and Milton, 2012). It is commonly used in a drug discovery setting to evaluate the developability of new molecule identities. To determine the TI, a range of metrics that reflect pharmacological and toxicological endpoints from *in vitro* or *in vivo* can be used (Muller and Milton, 2012). The use of these metrics depends on the stage of drug discovery and often evolves from *in vitro* target selectivity to *in vivo* preclinical species efficacy and safety over the journey of a drug discovery project. Before a drug candidate enters the clinic, a commonly adopted approach is to calculate systemic exposure multiples using the no observable adverse effect level or highest nonseverely toxic dose in the case of oncology, determined from animal toxicology evaluations, versus the anticipated human exposure for intended pharmacological activity. The approach provides a quantitative assessment of the TI and can be determined based on the drug peak concentration (C_max_) or the area under the curve (AUC). However, there are several limitations to the exposure-centric approach. The approach does not take into consideration of species differences in pharmacodynamic (PD) or toxicological responses, which is often true for biotherapeutic agents. A biologic drug candidate for a human disease may not cross-react with an animal target and a surrogate drug, which differs from the human drug candidate in target binding affinity or pharmacological activity, needs to be studied for efficacy or safety in the preclinical setting. Even if the human drug candidate cross-reacts with the target in preclinical species, it may have a different affinity to animal target than that in humans or suffers immunogenicity issues. Also, there may exist species differences in the sensitivity of toxicological responses (Attarwala, 2010; Chaikin, 2017). These challenges render the exposure-based TI less relevant to the assessment of human safety. Additionally, the human exposure prediction generally carries uncertainty because of limitations in experimental data and prediction methodologies. It adds ambiguity to the TI determination. Furthermore, the exposure-based TI calculations assume that systemic exposure is relevant to efficacy and safety, which may not be true when the underlying mechanism of toxicity is not well understood. Because of these considerations, it may be ideal to determine the TI in the same preclinical species, particularly if the pharmacology and toxicology information in that species is proven to be relevant to humans.

Interleukin-10 (IL-10) is a pleiotropic dimeric cytokine produced by many immune cells and carries both anti- and pro-inflammatory functions (Moore et al., 2001). Because of its pro-inflammatory effects on the expansion and activation of primed CD8^+^ T cells and natural killer cells, IL-10 has been explored as an immuno-oncologic agent for treating cancer (Ni et al., 2020). Owing to the short terminal half-life (t_1/2_, 2.3–3.7 h) of a recombinant human IL-10 (hIL-10) in the clinic (Huhn et al., 1996), PEGylation and antibody conjugation were used as a method for extending its systemic t_1/2_ (Ni et al., 2020). In this work, we examined the use of fragment crystallizable (Fc) fusion as an approach to extend the IL-10 t_1/2_. Mutations were introduced in the Fc domains to eliminate or reduce antibody-dependent cellular cytotoxicity and complement-dependent cytotoxicity. The C-terminus of the Fc domain was then fused with wild-type IL-10 through a glycine-serine rich polypeptide linker to retain the IL-10 activity. Although hIL-10 cross-reacts with the mouse IL-10 (mIL-10) receptor alpha, there existed an immunogenicity concern on the antidrug antibody (ADA) formation that affects pharmacokinetics (PK). As a result, we opted to construct a mouse Fc-fused mIL-10 (mFc-mIL-10) for pharmacology evaluations in mice. The binding affinity of mFc-mIL-10 to the mIL-10 receptor alpha was 3.2 nM, which was similar to that (2.9 nM) of a human Fc-fused hIL-10 (hFc-hIL-10) to the mouse receptor. Additionally, using mouse CD3^+^ T cells, the *in vitro* cellular EC_50_ of mFc-mIL-10 for the phosphorylation of signal transducer and activator of transcription 3 was 0.44 nM, comparable to that (0.55 nM) of hFc-hIL-10 determined in the same assay. The antitumor efficacy of mFc-mIL-10 was evaluated in mouse syngeneic tumor models as a single agent or in combination with a mouse antibody against the mouse programmed death-1 receptor (mPD-1). Blockade of the PD-1 pathway was known to enhance T-cell activation and restore T-cell effector function (McDermott and Atkins, 2013). Together with the immune agonist activity of IL-10, the combination of the anti-mPD-1 and mFc-mIL-10 treatment was expected to be synergistic and enhance antitumor immunity. Additionally, the expansion of CD8^+^ T cells following the IL-10 treatment led to the secretion of interferon gamma (IFNγ) and the subsequent production of interleukin-18 (IL-18) by antigen-presenting cells in a positive feedback loop, where IL-18 further augmented the IFNγ secretion and T-cell proliferation (Iwai et al., 2008). Clinically, the IL-18 fold-induction correlated with the antitumor effect of PEGylated IL-10 (Naing et al., 2018). Therefore, the IL-18 induction may serve as a PD biomarker after the IL-10 treatment and correlate with antitumor efficacy in mice.

For toxicity testing, the cynomolgus monkey was selected as the species for studying hFc-hIL-10. The prior preclinical and clinical safety experience showed that anemia and thrombocytopenia were common hematological adverse events (AEs) associated with the recombinant hIL-10 treatment in the clinic and these AEs were manifested in monkeys but not in mice (Fedorak et al., 2000; Schreiber et al., 2000; Rosenblum et al., 2002). Also, the hFc-hIL-10 binding affinity to the cynomolgus monkey and human IL-10 receptor alpha was determined to be 0.3 and 0.6 nM, respectively, justifying the use of cynomolgus monkeys for evaluating the safety profile of hFc-hIL-10.

With efficacy readouts generated in tumor-bearing mice and safety data obtained from cynomolgus monkeys, it was necessary to integrate the pharmacology and toxicology information to gauge the TI for developability assessment. Specifically, the objective of the work was to determine the TI of hFc-hIL-10 in the cynomolgus monkeys that exhibited toxicity relevant to human safety, whereas mice were insensitive to the IL-10-related toxicity. To achieve this goal, we applied PK/PD modeling as a tool for quantitative data integration. For mouse data, efforts were made to understand the relevance of the mouse IL-18 (mIL-18) induction as a PD biomarker to drug exposure and efficacy. For monkey data, PK/PD relationships were established on the induction of cynomolgus monkey IL-18 (cIL-18) and toxicological endpoints measured as anemia and thrombocytopenia. With the establishment of PK/PD relationships, we then used the induction of IL-18 to bridge efficacy from mice to monkeys and coupled it with monkey toxicology data to determine the TI of hFc-hIL-10 in the same species. Additionally, a PK-based TI determination with affinity adjustments between species was obtained. Collectively, the work was critical to informing the developability of hFc-hIL-10 and demonstrated the value of model-based approaches in drug discovery.

## 2 Materials and Methods

### 2.1 Materials and Reagents

mFc-mIL-10 and hFc-hIL-10 (molecular mass: 91,004 and 90,815 Da, respectively) were produced at Bristol Myers Squibb, Princeton, NJ. A biotinylated mouse anti-human immunoglobin G (IgG) Fc monoclonal antibody (mAb) and a mouse anti-mPD-1 IgG1 mAb with an inert effector function by a single amino acid substitution of aspartic acid to alanine at codon 265 (D265A) were also generated at Bristol Myers Squibb, Redwood City, CA. A rat anti-mIL-10 biotinylated antibody, a rat anti-hIL-10 antibody, a goat anti-mouse IgG antibody labeled with Alexa Fluor 647, and a mouse anti-monkey IgG labeled with horseradish peroxidase were purchased from Southern Biotech (Birmingham, AL). A mouse IL-18 (mIL-18) capture antibody and a biotinylated detection antibody were both purchased from R&D Systems (Minneapolis, MN). All other reagents were obtained from commercial sources in analytical grade.

### 2.2 Mouse Studies

The studies were conducted according to the study protocols approved by the Bristol Myers Squibb Institutional Animal Care and Use Committee. Animals were acclimated for 7–17 days before the studies and had free access to food and water ad libitum with a 12-h light/dark cycle.

#### 2.2.1 Mouse Antitumor Efficacy Studies With Mouse IL-10 Mouse Fc-Fusion Protein

Efficacy studies with mFc-mIL-10 were conducted in the MC38 and CT26 mouse syngeneic tumor models. Briefly, tumor cells (1 million) suspended in sterile Hanks’ balanced salt solution were subcutaneously implanted on the right flank of animals. Dosing was initiated typically 7 days after reaching a mean tumor volume of 100 mm^3^ and randomization of animals into treatment groups. Mice were weighed immediately post-randomization (before administration of treatment) and at least twice weekly through Day 28. Tumor volume was determined by measuring the length (mm) and width (mm), width defined as the smaller of the two measurements, from each tumor by a digital caliper and was calculated using the formula: tumor volume (mm^3^) = 0.5 × length × width^2^. The tumor volume throughout the study was recorded twice weekly. Mice that reached a volume of ≥1,000 mm^3^ for two consecutive measurements were euthanized due to the high tumor burden. Efficacy in this study was assessed by complete regression of the tumor which was defined as a tumor reaching a size of 0 mm^3^ for at least two consecutive measurements.

In the MC38 model (female C57BL6 mice, ∼20 g, Charles River Laboratories, Wilmington, MA), the antitumor efficacy of mFc-mIL-10 was evaluated as a single agent in two studies. The drug formulated in phosphate-buffered saline (PBS) was given as a single dose intraperitoneally (IP) to mice at doses of 0.1, 0.3, 1, 3, and 10 mg/kg, with 10 mice per dose group.

In the CT26 model (female BALB/c mice, ∼20 g, Envigo, Indianapolis, IN), three antitumor efficacy studies were performed with mFc-mIL-10 in combination with a mouse anti-mPD-1 mAb. The anti-mPD-1 mAb was administered every 4 days at 10 mg/kg IP for 3 doses, while mFc-mIL-10 was given as a single IP dose. The doses of mFc-mIL-10 were different among the three studies. In the first study, mFc-mIL-10 doses were 0.1, 0.3, and 1 mg/kg. They were 0.1 and 0.3 mg/kg in the second study and 0.03, 0.1, and 0.3 mg/kg in the third study. For efficacy evaluations, 8 or 10 mice were studied at each dose level along with an isotype control group and an anti-mPD-1 mAb group.

In these efficacy studies, blood samples were obtained for determining mFc-mIL-10 concentrations, with detailed information (type of samples, sample volume, time points, number of animals, serial versus composite, and sampling method) summarized in Supplementary Table S1.

#### 2.2.2 Mouse Pharmacokinetic/Pharmacodynamic Study on Mouse IL-18 Induction by Mouse IL-10 Mouse Fc-Fusion Protein in the CT26 Syngeneic Tumor Model

Female BALB/c mice (∼20 g, Envigo, Indianapolis, IN) bearing CT-26 syngeneic tumors in a size of 100 mm^3^ were used in the study. Tumor-bearing mice were randomized and divided into 6 groups with 6 mice in each group. mFc-mIL-10 formulated in PBS was dosed IP to two groups of mice at the doses of 0.1 and 0.3 mg/kg, respectively. Two additional groups of mice received mFc-mIL-10 at the doses of 0.1 and 0.3 mg/kg, respectively, along with the treatment of the anti-mPD-1 mAb that was dosed IP at 10 mg/kg every 4 days for 3 doses. Additionally, there were two control groups: one group of mice received an isotype control antibody, and the other group was given the anti-PD-1 mAb at 10 mg/kg every 4 days for 3 doses. In the study, blood samples were harvested for determining the mFc-mIL-10 and mIL-18 concentrations, with detailed information available in Supplementary Table S1.

### 2.3 Cynomolgus Monkey Studies

The studies were conducted according to the study protocols approved by the Bristol Myers Squibb Institutional Animal Care and Use Committee. Animals had a pretest period of at least 10 days before the studies and had free access to food and water ad libitum with a 12-h light/dark cycle.

#### 2.3.1 Monkey Single-Dose Pharmacokinetic Study With Human IL-10 Human Fc-Fusion Protein

hFc-hIL-10 was administered as a slow intravenous (IV) bolus injection (0.25 ml/kg) at the doses of 0.005, 0.05, and 0.5 mg/kg (*n* = 1 at each dose) *via* the saphenous vein to protein therapeutic-naive male cynomolgus monkeys (2.5–4.5 kg, Buckshire Corporation, Perkasie, PA). The drug was formulated in a solution that contained 20 mM histidine, 260 mM sucrose, 0.05 mM diethylenetriaminepentaacetic acid (DTPA), and 0.05% Tween 80 at pH 7.4. Blood samples were collected for evaluating hFc-hIL-10 concentrations, with the detailed information listed in Supplementary Table S1.

#### 2.3.2 Monkey Repeat-Dose Study With Human IL-10 Human Fc-Fusion Protein for Evaluation of Pharmacokinetics, Cynomolgus Monkey IL-18 Induction, Hematological Changes, and Antidrug Antibody Response

hFc-hIL-10 was administered IV every 2 weeks for three doses to protein therapeutic-naive cynomolgus monkeys (2.5–4.5 kg, Buckshire Corporation, Perkasie, PA). The drug was prepared in a dosing solution that contained 20 mM Tris, 260 mM sucrose, 0.05 mM DTPA, and 0.05% Tween 80 at pH 7.4 and was given as a slow bolus injection (0.25 ml/kg) *via* the saphenous vein. Each dose group had 3 monkeys (1 male and 2 females at 0.06 mg/kg; 2 males and 1 female at 0.18 mg/kg) along with a group of animals dosed with the vehicle. In the study, blood samples were obtained for determining hFc-hIL-10 concentrations, cIL-18 concentrations, hematological changes (hematocrit and platelet counts), and ADA responses. The detailed information is summarized in Supplementary Table S1.

### 2.4 Sample Analysis

Quantification methods for mFc-mIL-10, mIL-18, hFc-hIL-10, cIL-18, ADA, platelet counts, and hematocrit are available in Supplemental Materials.

### 2.5 Data Analysis

Data are expressed as mean ± standard deviation (SD). For diluted blood samples collected in mice, a theoretical dilution factor of 17.36 was used for correcting the drug levels to undiluted plasma drug concentrations (Joyce et al., 2014). The validity of the approach was confirmed by the experimental observations (Yang et al., 2022).

PK/PD data modeling and simulation were conducted using SAAM II (v2.3.1.1, The Epsilon Group, Charlottesville, VA). The selections of the weighting function were based on the weighted residual plots. The goodness of fit was assessed by the minimization of the objective function (expressed as—2 times the log-likelihood function), Akaike information criterion (AIC), Schwarz-Bayesian information criterion (SBIC), visual inspection of the fitting, and the precision of the parameters estimated.

#### 2.5.1 Pharmacokinetic/Pharmacodynamic Modeling of Mouse IL-18 Induction Data in Mice

**FIGURE 1:**
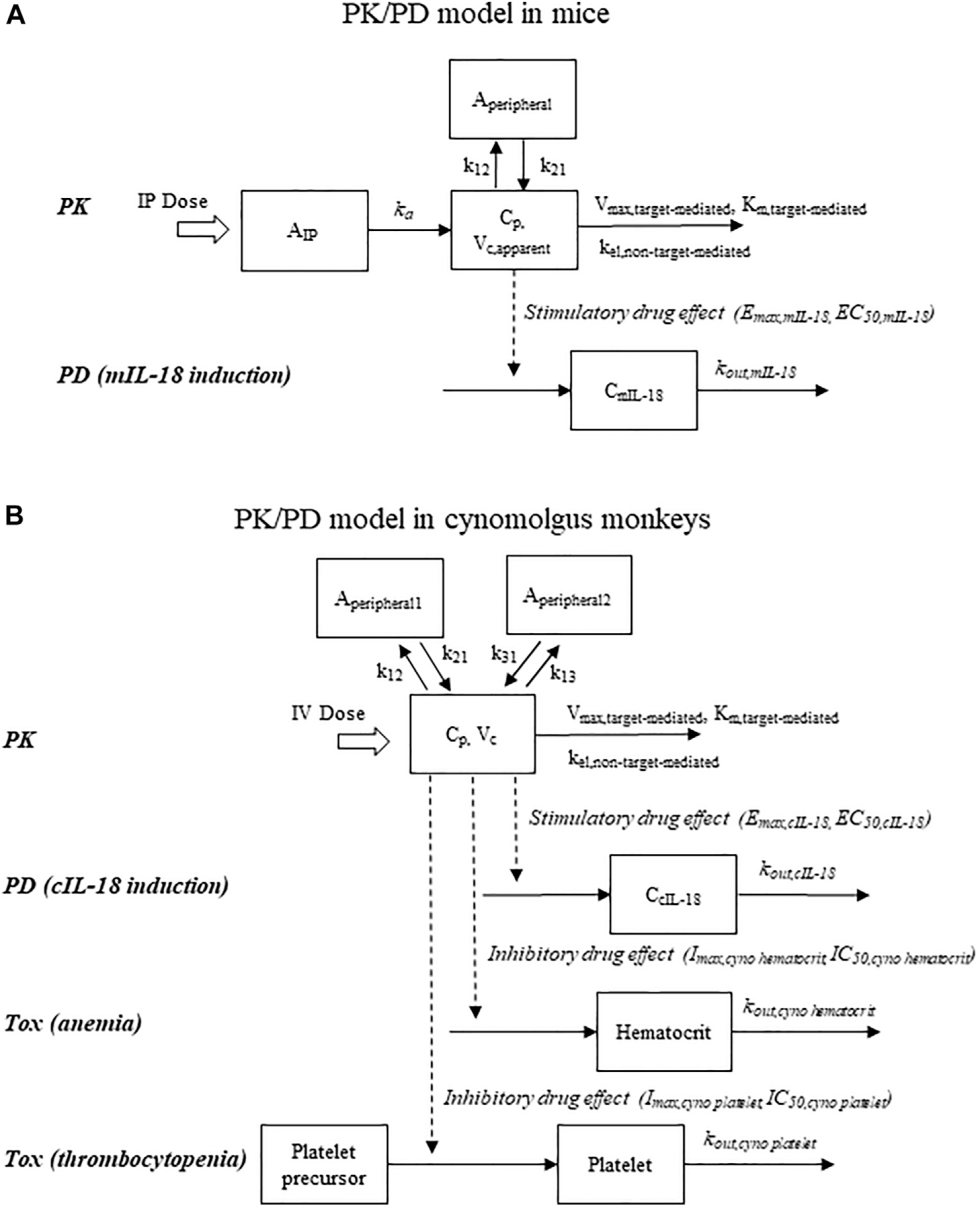
Schematic representations of pharmacokinetic/pharmacodynamic models that describe drug concentrations, pharmacodynamic response (IL-18 induction), and hematological adverse events (reductions in hematocrit and platelet counts). **(A)** Mouse model; **(B)** Cynomolgus monkey model. To account for the time delay observed with the IL-18 induction in mice, a time lag in drug administration was incorporated into the mouse model.

The average PK and mIL-18 data from the mouse PK/PD study described in Section 2.2.2 were fitted sequentially, with the model scheme shown in Figure 1A. A two-compartment model incorporated with Michaelis-Menten elimination kinetics and first-order absorption previously described for mFc-mIL-10 (Yang et al., 2022) was used to fit the PK data after IP administration. The differential equations are shown below.

$$\frac{dA_{IP\;}}{dt}=-k_{a}\times A_{IP}$$ (1)

$$V_{c,apparent}\frac{dC_{P}}{dt}=\;k_{a}\times A_{IP}+k_{21}\times A_{peripheral}-\left(k_{12}+k_{el,non-t\arg et-mediated}+\frac{V_{\max,\;t\arg et-mediated}}{k_{m,t\arg etmediated}\times V_{c,apparent}+V_{c,apparent}\times C_{p}}\right)\times V_{c,apparent}\times C_{p}$$ (2)

$$\frac{dA_{peripheral}}{dt}=k_{12}\times V_{c,apparent}\times C_{p}-k_{21}\times A_{peripheral}$$ (3)

where A_IP_ is the amount of the drug at the IP absorption site; A_peripheral_ is the drug amount in the peripheral compartment; C_p_ is the drug concentration in the central compartment; V_c,apparent_ is the apparent volume of distribution in the central compartment after IP administration; k_a_ is the absorption rate constant; k_12_ and k_21_ are the transfer rate constants between the central and peripheral compartments; k_el,non-target-mediated_ is the non-target-mediated first-order elimination rate constant; V_max,target-mediated_ is the maximum elimination rate mediated by a target; K_m,target-mediated_ is the binding affinity to the target. For the PK data, owing to only three-time points for drug concentrations available in the mIL-18 induction study, the mFc-mIL-10 PK parameters obtained from other IV PK and IP efficacy studies (Yang et al., 2022) were used as the fixed values. The only exception was the V_c,apparent_. It was fitted to the data as a scalar to account for the apparent exposure differences between the studies. The approach allowed an adequate description of the mFc-mIL-10 PK profiles observed in the study, which was essential before modeling the mIL-18 induction data.

To model the mIL-18 induction data, an indirect response model with drug stimulatory effects on the rate of mIL-18 production was employed. The delay in the mIL-18 induction following the mFc-mIL-10 treatment was modeled by shifting the time of drug administration by 72 h to account for the T-cell expansion, INFγ secretion, and subsequent mIL-18 production by antigen-presenting cells (Naing et al., 2018). The approach was adopted because transit compartment models cannot describe the sharp decline of mIL-18 levels. Furthermore, the objective function, AIC, and SBIC using the transit compartment models were higher than those using the time point delay model.

The interaction between mFc-mIL-10 and the mIL-10 alpha receptor was described using an Emax model and the effect signal was then applied as a forcing function for the mIL-18 production. The differential equation is displayed below:

$$\begin{array}{c} \frac{dC_{mIL-18\;}}{dt}=\left(C_{mIL-18,baseline,time\;}+\frac{E_{\max,mIL-18}\;x\;C_{p}}{EC_{50,mIL-18}\;+\;C_{p}}\right)\times k_{out,mIL-18}\\ -k_{out,mIL-18}\times C_{mIL-18\;} \end{array}$$ (4)

where C_mIL-18_ is the mIL-18 concentration; C_mIL-18,baseline,time_ is the mIL-18 baseline level in the absence of the mFc-mIL-10 treatment; E_max,mIL-18_ is the maximum effect produced by mFc-mIL-10; EC_50,mIL-18_ is the plasma mFc-mIL-10 concentration corresponding to the half of the E_max,mIL-18_; k_out,mIL-18_ is the turnover rate constant of mIL-18. The turnover t_1/2_ of mIL-18 was calculated as 0.693/k_out,mIL-18_.

The observed mIL-18 data revealed that the magnitude of the mIL-18 induction in the anti-mPD-1 mAb combination group was higher than that in the monotherapy group. Mechanistically, PD-1 is a coinhibitory receptor on T cells and plays an important immunoregulatory role in the T cell activation, differentiation, and effector function (Freeman et al., 2000; Jubel et al., 2020). An anti-PD-1 blocking mAb enhances T cell activation and restores T-cell effector function (McDermott and Atkins, 2013). With more activated T cells, more IL-10 receptor alpha is expressed in T cells (Emmerich et al., 2012; Naing et al., 2018). Endogenous IL-10 production is also increased upon anti-PD-1 treatment (Lamichhane et al., 2017). Both aspects lead to more production of IFNγ and further induction of IL-18 by myeloid cells. Additionally, IL-10 is known to drive the proliferation and expansion of LAG-3^+^PD-1^+^CD8^+^ T cells (Naing et al., 2018), which in turn enhances the response of the anti-PD-1 treatment and creates a possible synergic interaction on the IL-18 induction. To describe the enhanced mIL-18 induction in the presence of the anti-mPD-1 mAb, the following equation was used:

$$\frac{dC_{mIL-18\;}}{dt}=\left(C_{mIL-18,baseline,time\;}+\frac{Fold_{anti-mPD-1}\times E_{\max,mIL-18}\;x\;C_{p}}{EC_{50,\mathrm{mouse}\;\mathrm{IL}-18}\;+\;C_{p}}\right)\times k_{out,mIL-18}-k_{out,mIL-18}\times C_{mIL-18\;}$$ (5)

where fold_anti-mPD-1_ represents the observed interaction on the IL-18 induction in the anti-mPD-1 mAb combination group, while the EC_50,mIL-18_ remained the same between the single-agent and combination groups.

In the isotype control and the anti-mPD-1 mAb monotherapy groups, a time-dependent increase in the mIL-18 level was observed, which did not correlate with the growth of tumor size. To account for the baseline change, a linear equation was applied to describe the C_mIL-18,baseline,time_ change with time in the control groups, using the equation shown below:

$$C_{mIL-18,baseline,time\;}=C_{mIL-18,baseline,predose\;}+slope_{mIL-18,baseline}\times time$$ (6)

where C_mIL-18,baseline,predose_ is the baseline mIL-18 level before the treatment; slope_mIL-18,baseline_ is the rate of mIL-18 change in the control groups over time.

Additionally, the existing mIL-18 data did not result in a reliable estimate in the k_out,mIL-18_. To facilitate parameter identification, the Bayesian estimation method available in SAAM II (Callegari et al., 2002) was used. Previously, the mIL-18 t_1/2_ after IP administration to mice was reported to be 16 h (Hosohara et al., 2002), which corresponded to the k_out,mIL-18_ of 0.043 h^−1^ (= 0.693/16 h). In humans, the %CV associated with the t_1/2_ of a human recombinant IL-18 was estimated to be ∼40% based on the median and the range reported in 24 subjects at the doses of 3 to 1,000 µg/kg (Robertson et al., 2006). With the information from mouse and human recombinant IL-18, the population mean of 0.043 h^−1^ and a standard deviation of 0.022 h^−1^ (50% CV) were applied as the prior information for estimating the k_out,mIL-18_ from the mIL-18 induction data.

At time zero, the following conditions exist:

$$A_{IP,\;t=0}=Dose_{IP}$$

$$C_{P,\;t=0}=0$$

$$A_{Periperial,t=0}=0$$

$$C_{mIL-18,\;baseline,time}=C_{mIL-18,baseline,predose}$$

where Dose_IP_ is the dose administered *via* the IP route. Additionally, the weighting function for fitting mouse PK and mIL-18 data was 1/y and equal weighting, respectively.

#### 2.5.2 Simulation of Mouse IL-18 Induction in Mouse Efficacy Studies

Based on the PK/PD model established, the extent of the mIL-18 induction following the mFc-mIL-10 treatment as a single agent or in combination with the anti-mPD-1 mAb in efficacy studies was simulated using SAAM II. The PK parameters used for the simulations were the values published for the drug concentration data observed in efficacy studies (Yang et al., 2022). These PK parameter values are listed in Table 1 except for the V_c,apparent_ (45 ml/kg) that was obtained after fitting the PK data observed in the MC38 and CT26 efficacy studies (Yang et al., 2022). The PD parameters used for the simulations were obtained after the PK/PD modeling of the mIL-18 induction data as described in Section 2.5.1. Additionally, for the simulations, no major strain difference was assumed in the mIL-18 induction between C57BL6 mice carrying the MC38 tumor and BALB/c mice bearing the CT26 tumor. The induction of the mIL-18 level was simulated at the single mFc-mIL-10 IP doses of 0.03, 0.1, 0.3, 1, 3, and 10 mg/kg for 5 weeks either as a single agent or in combination with the anti-mPD-1 mAb. The simulated results were the point estimates using the model parameters without considering the uncertainty (i.e., estimation errors) associated with the parameters. The AUC values of mIL-18 were calculated using the linear trapezoidal rule. The fold of mIL-18 C_max_ induction was calculated by comparing the simulated mIL-18 Cmax with the baseline level at the same time point. The fold of mIL-18 AUC induction over the intervals of 2, 3, and 4 weeks was estimated by the simulated mIL-18 AUC vs. that in the control group (isotype control for monotherapy and the anti-mPD-1 mAb for the combination therapy) to mimic the clinical dosing regimens of once every 2, 3, and 4 weeks (Q2W, Q3W, and Q4W).

**TABLE 1:** Pharmacokinetic and pharmacodynamic parameters estimated for mFc-mIL-10 after intraperitoneal administration to mice bearing CT26 syngeneic tumors at doses of 0.1 and 0.3 mg/kg either as a single agent or in combination with mouse anti-mouse PD-1 monoclonal antibody^a^.

Parameter	Value estimated (mean ± standard error)
**Pharmacokinetics^b^**	
V_c,apparent_ (L/kg)	3.6 × 10^−2^ ± 7.8 × 10^−4^ ^c^
k_12_ (1/h)	2.13 × 10^−1^ (fixed)
k_21_ (1/h)	2.09 × 10^−1^ (fixed)
k_el,non-target-mediated_ (1/h)	1.9 × 10^−2^ (fixed)
K_m,target-mediated_ (nM)	3.2 (fixed)
V_max,target-meidated_ (nmol/kg/h)	9.8 × 10^−3^ (fixed)
k_a_ (1/h)	2.5 × 10^−1^ (fixed)
**Pharmacodynamics (mIL-18 induction)**	
C_mIL-18,baseline,predose_ (pg/ml)	113 ± 4.4
Slope_mIL-18_,_baseline_ (pg/ml/h)	1.5 × 10^−1^ ± 2.3 × 10^−2^ (monotherapy)
	1.9 × 10^−1^ ± 1.7 × 10^−2^ (combination with anti-mPD-1)
E_max,mIL-18,monotherapy_ (pg/ml)	126 ± 22
Fold_anti-mPD-1_	4.4 ± 0.73
EC_50,mIL-18_ (nM)	2.4 ± 0.56
k_out,mIL-18_ (1/h)	6.4 × 10^−2^ ± 2.2 × 10^−2^

a The dose of the anti-mouse PD-1 mAb was 10 mg/kg given once every 4 days for 3 doses.

b Because of 3-time points available for drug concentrations in the study, only the V_c,apparent_ was fitted to the pharmacokinetic data, with the rest of the pharmacokinetic parameters obtained from other datasets and used as the fixed values (Yang et al., 2022).

c The V_c,apparent_ at 0.1 mg/kg in combination with the anti-mPD-1 mAb was estimated to be 8.4 × 10^−2^ ± 3.3 × 10^−3^ L/kg (fitting performance is available in Supplementary Table S2).

#### 2.5.3 Pharmacokinetic/Pharmacodynamic Modeling of Cynomolgus Monkey IL-18 Induction, Platelet Count, and Hematocrit Data in Cynomolgus Monkeys

Figure 1B shows the model structure used to describe the PK, cIL-18, platelet count, and hematocrit data following IV administration of hFc-hIL-10 to cynomolgus monkeys. The average PK data from the single- and repeat-dose studies were fitted first, followed by modeling the average cIL-18, platelet count, or hematocrit data. The PK model was a 3-compartment model, with both target- and non-target-mediated elimination incorporated in the model. The differential equations after an IV bolus dose are shown below:

$$V_{c}\frac{dC_{P}}{dt}=\;k_{21}\times A_{peripheral1}+k_{31}\times A_{peripheral2}-\left(k_{el,non-t\arg et-mediated}+k_{12}+k_{13}+\frac{V_{\max,\;t\arg et-mediated}}{K_{m,t\arg etmediated}\times V_{c}+V_{c}\times C_{p}}\right)\times V_{c}\times C_{p}$$ (7)

$$\frac{dA_{peripheral1}}{dt}=k_{12}\times V_{c}\times C_{p}-k_{21}\times A_{peripheral1}$$ (8)

$$\frac{dA_{peripheral2}}{dt}=k_{13}\times V_{c}\times C_{p}-k_{31}\times A_{peripheral2}$$ (9)

where A_peripheral1_ and A_peripheral2_ are the drug amounts in the peripheral compartments 1 and 2, respectively; k_12_, k_21_, k_13_, and k_31_ are the transfer rate constants between the central and peripheral compartments, respectively.

To model the cIL-18 induction data, the indirect response model same as what was applied to the mIL-18 data was used, except that no lag time was needed. One monkey at 0.18 mg/kg was not included in the data analysis, because of a much higher predose baseline than the rest of the animals. Additionally, a time-dependent decrease in the cIL-18 levels of the vehicle control group was correlated linearly with time (*R* ^2^ = 0.70, with *p* < 0.05 for the slope), which was incorporated in the model. The equations are shown below:

$$\frac{dC_{cIL-18\;}}{dt}=\left(C_{cL-18,baseline,time\;}+\frac{E_{\max,cIL-18}\;x\;C_{p}}{EC_{50,cIL-18}\;+\;C_{p}}\right)\times k_{out,cIL-18}-k_{out,cIL-18}\times C_{cIL-18\;}$$ (10)

$$C_{cIL-18,\mathrm{baseline},time\;}=C_{cIL-18,baseline,predose\;}-slope_{cIL-18,\;\mathrm{baseline}}\times time$$ (11)

where C_cIL-18,baseline,time_ is the cIL-18 baseline level in the absence of the hF-hIL-10 treatment; C_cIL-18,baseline,predose_ is the cIL-18 baseline level before drug treatment at time zero; E_max,cIL-18_ is the maximum drug effect; EC_50,cIL-18_ is the plasma drug concentration corresponding to the half of the E_max,cIL-18_; k_out,cIL-18_ is the turnover rate constant of cIL-18; slope_cIL-18,baseline_ is the rate of cIL-18 change in the control groups over time. The turnover t_1/2_ of cIL-18 was calculated as 0.693/k_out,cIL-18_. Additionally, to improve the precision of the parameter estimates, the Bayesian estimation method in SAAM II (Callegari et al., 2002) was used for estimating the EC_50,cIL-18_ by initially fitting the data and then applying the parameter estimates as a population mean with a 50% CV for the final model fitting.

To describe the platelet count data in cynomolgus monkeys, an indirect response model with a precursor was used, with inhibitory drug effects assumed to be on the production of platelets. The precursor model was necessary because of the rebounding effect of the platelets that went above the predose level after drug treatment. There was also no apparent time-dependent change in the platelet counts in the vehicle control group. The differential equations are displayed below:

$$\frac{dC_{cyno\;platelet\;precursor\;}}{dt}=C_{cyno\;platelet\;precursor,predose}\times k_{out,cyno\;platelet}-k_{out,cyno\;platelet}\times \left(1-\frac{I_{\max,cyno\;platelet}\;x\;C_{p}}{IC_{50,cyno\;platelet}\;+\;C_{p}}\right)\times C_{cyno\;platelet\;precursor}$$ (12)

$$\frac{dC_{cyno\;platelet\;}}{dt}=k_{out,cyno\;platelet}\times \left(1-\frac{I_{\max,cyno\;platelet}\;x\;C_{p}}{IC_{50,cyno\;platelet}\;+\;C_{p}}\right)\times C_{cyno\;platelet\;precursor}-k_{out,cyno\;platelet}\times C_{cyno\;platelet\;}$$ (13)

where C_cyno platelet precursor_ and C_cyno platelet_ are the concentrations of platelet precursor and platelet, respectively; C_cyno platelet precursor,predose_ is the predose level of the platelet precursor in cynomolgus monkeys; I_max,cyno platelet_ is the maximum inhibitory effect produced by hFc-hIL-10 and fixed at 100%; IC_50,cyno platelet_ is the plasma drug concentration corresponding to the half of the I_max,cyno platelet_; and k_out,cyno platelet_ is the turnover rate constant of the platelets in cynomolgus monkeys. The turnover t_1/2_ of platelet counts was calculated as 0.693/k_out,cyno platelet_.

To fit the hematocrit data observed, an indirect response model was employed, with inhibitory drug effects affecting the production of red blood cells. Also, the hematocrit data were relatively constant in the vehicle control group. The differential equation is displayed below:

$$\frac{dC_{cyno\;hematocrit\;}}{dt}=C_{cyno\;hematocrit,predose}\times k_{out,cyno\;hematocrit}\times \left(1-\frac{I_{\max,cyno\;hematocrit}\;x\;C_{p}}{IC_{50,cyno\;hematocrit}\;+\;C_{p}}\right)-k_{out,cyno\;hematocrit}\times C_{cyno\;hematocrit}$$ (14)

where C_cyno hematocrit_ is the hematocrit value in percentage; C_cyno hematocrit,predose_ is the predose, baseline value of hematocrit; I_max,cyno hematocrit_ is the maximum drug effect and fixed at 100%; IC_50,cyno hematocrit_ is the plasma drug concentration corresponding to the half of the I_max,cyno hematocrit_; and k_out,cyno hematocrit_ is the turnover rate constant of red blood cells in cynomolgus monkeys. The turnover t_1/2_ of red blood cells was calculated as 0.693/k_out,cyno hematocrit_.

At time zero, the following conditions exist:

$$C_{P,\;t=0}=\frac{Dose_{IV}}{V_{c}}$$

$$A_{Periperial1,t=0}=0$$

$$A_{Periperial2,t=0}=0$$

$$C_{cIL-18,t=0}=C_{cIL-18,predose}$$

$$C_{cyno\;platelet,t=0}=C_{cyno\;platelet,predose}$$

$$C_{cyno\;platelet\;percursor,t=0}=C_{cyno\;platelet\;precursor,predose}=C_{cyno\;platelet,predose}$$

$$C_{cyno\;hematocrit,t=0}=C_{cyno\;hematocrit,predose}$$

where Dose_IV_ is the dose administered *via* the IV route and C_cyno platelet,predose_ is the predose level of platelets. Based on the model structure, the predose level of platelet precursor needed to be the same as that of platelets to maintain a constant baseline in the model.

Additionally, because of the ADA formation that affected the PK after the 2nd and 3rd dose in the repeat-dose study, a dose-reduction factor was introduced to account for lower drug exposure. The use of the dose reduction factor in the model was necessary because ADA measurements were qualitative (Krishna and Nadler, 2016). Specifically, the drug in the samples competed with the drug as a capturing agent in the ADA assay and led to lower ADA titers at higher drug doses, even though the ADA effect on PK was more pronounced at higher doses. Consequently, the existing ADA data cannot be used to build a mechanistic model to account for the drug exposure loss. Using the dose reduction factor, the initial condition for the C_p,t=0_ after the 2nd and 3rd doses was equal to:

$$C_{P,\;t=0,\;2nd\;or\;3rd\;dose}=\frac{Dose_{IV}}{Dose\;reduction\;factor\times V_{c}}.$$

Given the variability observed in the ADA formation, individual dose reduction factors were required between the 2nd and 3rd dose and at different dose levels. The initial value for the dose reduction factor was estimated by comparing the C_max_ after the 1st dose versus that after the 2nd or 3rd dose. Because of insufficient data points available after the 2nd or 3rd dose, the Bayesian estimation method available in SAAM II (Callegari et al., 2002) was used to fit the observed data and estimate the final value by assuming the initial value as the population mean and a CV of 50%.

The weighting function for fitting cynomolgus monkey PK data was 1/y^2^, and equal weighting was used for modeling cIL-18, platelet count, and hematocrit data.

#### 2.5.4 Projection of Efficacious and Maximum Tolerated Doses in Cynomolgus Monkeys

The efficacious dose in cynomolgus monkeys was projected by achieving the cIL-18 AUC fold-induction same as that in mice where a complete tumor regression was observed in 80% of mice in efficacy studies. To project the efficacious dose in monkeys, the following assumptions were made: 1) no ADA effect on drug exposure and 2) no time-dependent change in the cIL-18 baseline. The assumptions represented the simplest and best-case scenario of the efficacious dose projected in monkeys and were sufficient for the TI determination. Using the PK/PD model established for the cIL-18 induction in cynomolgus monkeys, the cIL-18 concentration-time data following the IV treatment of hFc-hIL-10 at a range of doses were simulated under the dosing regimens of Q2W, Q3W, and Q4W. The cIL-18 baseline level used in the simulation was the median value (13 pg/ml) observed at predose in the study. The value was selected because it better reflected the central tendency of the cIL-18 predose level than the model-estimated result. The AUC values of cIL-18 were calculated using the linear trapezoidal rule, and the fold-induction in the cIL-18 AUC was obtained by comparing the values between the treatment and control groups. In addition to the PD-based method, a PK-based approach was employed for the efficacious dose projection in cynomolgus monkeys. Under the dosing regimens of Q2W, Q3W, and Q4W, the monkey efficacious doses were projected by targeting the steady-state AUC equal to the binding affinity-corrected mouse efficacious AUC from time 0 to infinity (AUC_tot_) following single IP injection. No ADA effect on drug exposure was also assumed. The mouse efficacious exposure was corrected linearly for the binding affinity difference between mice and monkeys (3.2 vs. 0.3 nM). The PK-based method projected two efficacious doses in cynomolgus monkeys because of differences in mouse efficacious doses observed between mono- and combination therapies. For both PD- and PK-based methods, simulations were conducted in SAAM II through the dose titration to determine the efficacious doses in cynomolgus monkeys for achieving targeted values.

To identify the maximum tolerated dose (MTD), simulations were also performed in SAAM II to examine hematological changes in cynomolgus monkeys at a dose range of 0.02–0.3 mg/kg under the Q2W, Q3W, and Q4W regimens. In the simulations, drug exposure was also assumed not altered upon repeat dosing. The MTD was defined as the dose that produced hematological AEs that were Grade 2 or less. The TI was calculated as the ratio of the MTD versus the projected efficacious dose. It is worth pointing out that the projected efficacious dose and MTD were the point estimates from the model without considering the estimation errors associated with model parameters.

## 3 Results

### 3.1 Mouse Studies

#### 3.1.1 Antitumor Efficacy and Drug Exposure in Mouse Syngeneic Tumor Models

**FIGURE 2:**
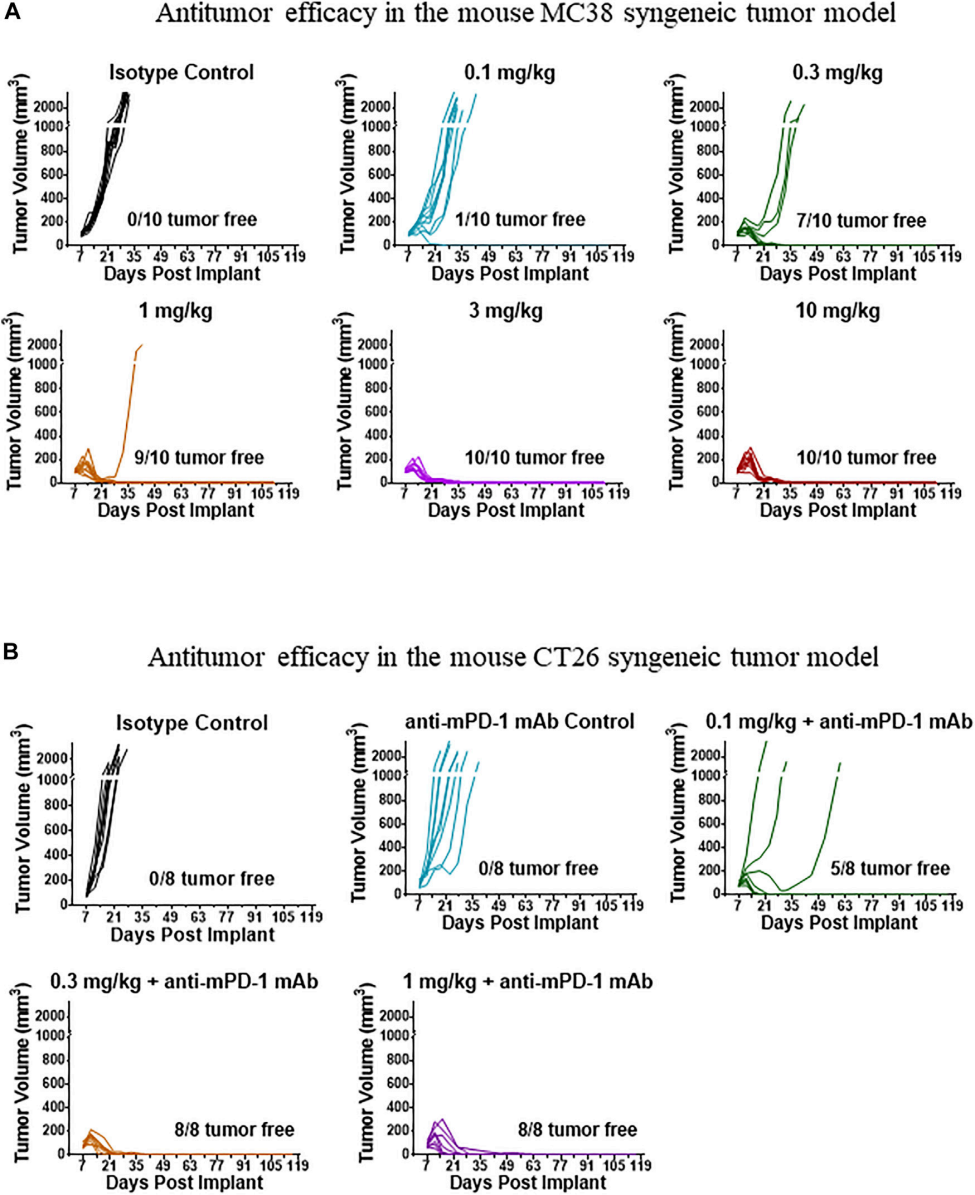
Representative tumor growth curves in tumor-bearing mice treated with mFc-mIL-10. **(A)** MC38 syngeneic tumor model where mFc-mIL-10 was given a single intraperitoneal injection at doses of 0.1, 0.3, 1, 3, and 10 mg/kg; **(B)** CT26 syngeneic tumor model where mFc-mIL-10 was injected intraperitoneally at a single dose of 0.1, 0.3, and 1 mg/kg in combination with a mouse anti-mouse programmed death-1 monoclonal antibody that was dosed at 10 mg/kg once every 4 days for 3 doses.

mFc-mIL-10 demonstrated robust antitumor efficacy in the MC38 and CT26 mouse syngeneic tumor models. The tumor growth curves of a representative study in the MC38 and CT26 models are shown in Figures 2A,B, respectively. In the MC38 model, the percentage of mice with complete tumor regression averaged from two studies at the single IP doses of 0.1, 0.3, 1, 3, and 10 mg/kg was 5%, 65%, 95%, 90%, and 90%, respectively, while there were no tumor-free mice in the isotype control group. In the CT26 model, mFc-mIL-10 was evaluated in combination with an anti-mPD-1 mAb in three studies. The percentage of mice that were tumor-free at the mFc-mIL-10 single IP doses of 0.03, 0.1, 0.3, and 1 mg/kg was 40%, 77%, 85%, and 100%, respectively. The percentage reported for 0.1 and 0.3 mg/kg was the value averaged from three studies and the percentage at 0.03 and 1 mg/kg was from one study. In comparison, averaging from three studies, the isotype control and anti-mPD-1 mAb monotherapy groups had 0% and 14% tumor-free mice, respectively.

In the efficacy evaluations, systemic exposure to mFc-mIL-10 was determined in four out of five studies. Across the dose range evaluated, mFc-mIL-10 exhibited nonlinear pharmacokinetics due to target-mediated drug disposition (TMDD). As shown by Yang et al. (2022), TMDD manifested to affect the PK of mFc-mIL-10 at pharmacologically active doses, presumably due to a reduction in the non-target-mediated clearance resulting from the neonatal Fc receptor-mediated recycling and molecular weight increases that reduced the renal clearance. Experimentally, the effect of TMDD on the mFc-mIL-10 PK was abolished by an anti-IL-10 receptor alpha-blocking antibody. As a result, a PK model described in Section 2.5.1 was developed to account for target- and non-target-mediated drug elimination and unify the PK data observed in efficacy studies (Yang, et al., 2022). Examples of drug exposure from efficacy studies and model-predicted results based on the PK parameters published by Yang et al. (2022) are shown in Supplementary Figures S1A, S1B.

#### 3.1.2 Pharmacokinetic/Pharmacodynamic Modeling of Mouse IL-18 Induction by Mouse IL-10 Mouse Fc-Fusion Protein

**FIGURE 3:**
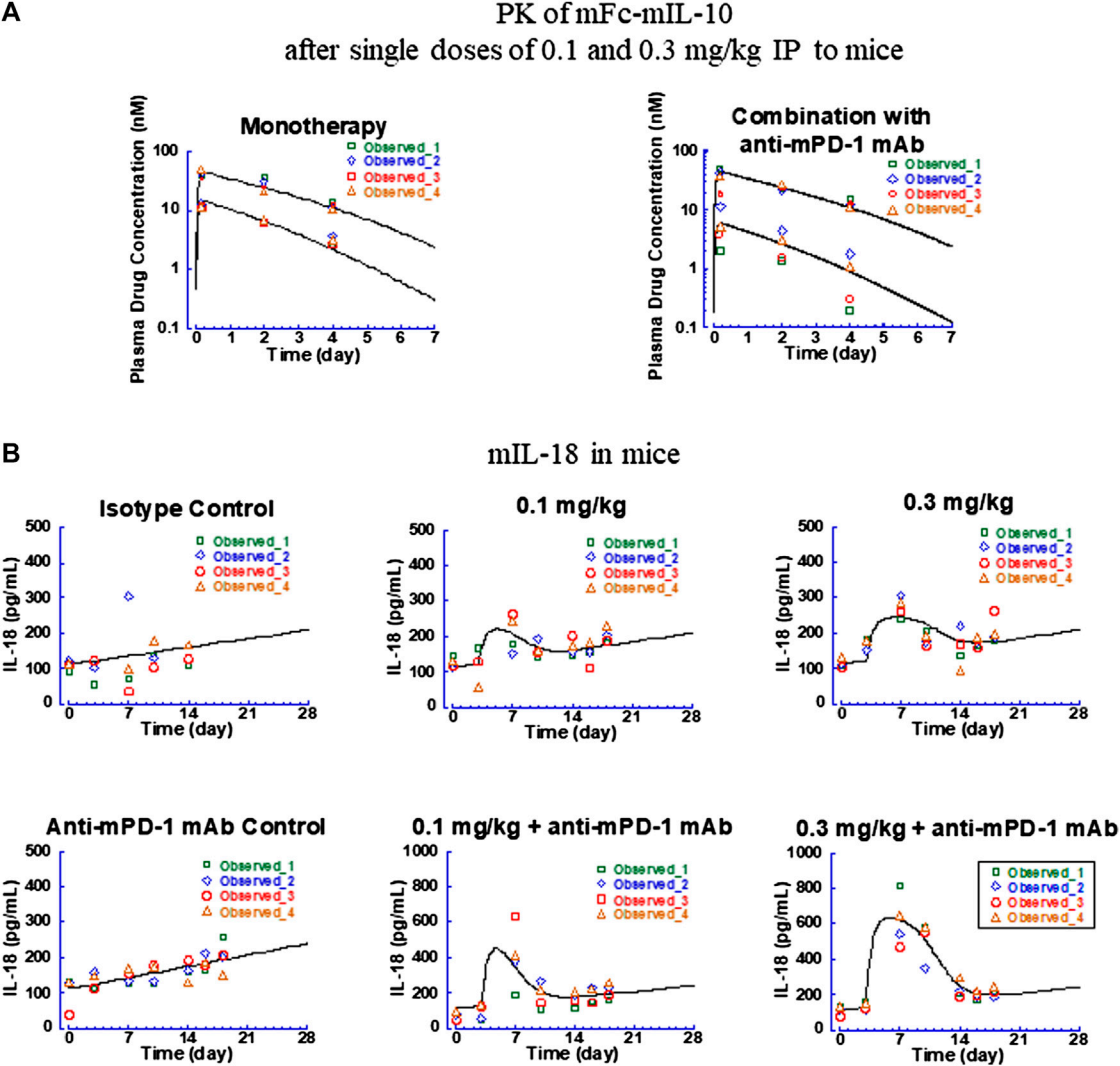
Fitted versus observed pharmacokinetic and IL-18 induction data in CT26 syngeneic tumor-bearing mice after intraperitoneal administration of mFc-mIL-10 at 0.1 and 0.3 mg/kg either as a single agent or in combination with a mouse anti-mouse programmed death-1 monoclonal antibody that was dosed intraperitoneally at 10 mg/kg every 4 days for 3 doses. **(A)** Pharmacokinetic profiles of mFc-mIL-10; **(B)** Time-course data of mouse IL-18 induction. Lines represent the model-fitted curves; symbols show individual observed data (*n* = 4 per time point). When data points are less than the number indicated, they are below the lower limit of quantitation for pharmacokinetic data or unavailable for mouse IL-18 data.

A PK/PD model was established to describe the induction of mIL-18 levels following IP administration of mFc-mIL-10 to mice bearing the CT26 tumor, either as a monotherapy or in combination with the anti-mPD-1 mAb. The key models evaluated and model performance measured as the objective function, AIC, and SBIC are summarized in Supplementary Table S2. The fitted versus observed PK and mIL-18 time course data are shown in Figures 3A,B, with the residual plots shown in Supplementary Figures S2A, S2B. The estimated PK and PD parameters are summarized in Table 1.

For the PK data, owing to only three-time points obtained for drug concentrations in the study, the PK parameters obtained with mFc-mIL-10 in other mouse efficacy and intravenous PK studies (Yang et al., 2022) were used as the fixed values. The only exception was the V_c,apparent_ which was fitted to the data as a scalar to account for the exposure differences between and within the studies. The V_c,apparent_ of mFc-mIL-10 estimated for monotherapy and the 0.3-mg/kg group in combination with the anti-mPD-1 mAb was 36 ml/kg, in agreement with the V_c,apparent_ of 45 ml/kg obtained from fitting the drug exposure data from efficacy studies (Yang et al., 2022). The exception was the 0.1-mg/kg combination group, where the V_c,apparent_ was estimated to be 84 ml/kg to best fit the observed drug concentrations. There is no known drug-drug interaction between mFc-mIL-10 and the anti-mPD-1 mAb. Specifically, in the same experiment, the exposure obtained from the combination group at 0.3 mg/kg was comparable to that of the monotherapy. Also, in efficacy studies across a wide dose range, no meaningful difference in drug concentrations was found between the monotherapy and combination groups (Yang et al., 2022). The plausible explanation was that there might be some issues associated with the dosing solution at 0.1 mg/kg in this experiment. Nevertheless, the approach of including two V_c,apparent_ was necessary to adequately describe the PK profiles of mFc-mIL-10 observed in the study before fitting the mIL-18 induction data (Figure 3A) and was supported by improvements in the objective function and information criteria measuring the model performance (Supplementary Table S2).

For the mIL-18 data, the PK/PD model captured the trend of the mIL-18 elevation produced by mFc-mIL-10 in the absence and presence of the anti-mPD-1 mAb (Figure 3B). An apparent increase in the IL-18 baseline over time was observed in the isotype control and anti-mPD-1 mAb monotherapy groups. A linear equation was used to account for time-dependent changes in the mIL-18 baseline, with the predose baseline level estimated to be 113 pg/ml and the slope of change over time in the monotherapy and combination groups determined to be 0.15 and 0.19 pg/ml/hour, respectively. From the modeling work, the E_max,mouse IL-18_ of mFc-mIL-10 as a single agent was estimated to be 126 pg/ml. When combined with the anti-mPD-1 mAb, there was a 4.4-fold increase in the E_max,mouse IL-18_ versus that of the single agent, indicating that the mIL-18 induction was potentiated by the anti-mPD-1 mAb. Mechanistically, the anti-mPD-1 mAb is known to lead to T-cell activations and subsequent interferon-γ secretion (Freeman et al., 2000; McDermott and Atkins, 2013), which further fuels the release of mIL-18 from antigen-presenting cells. The EC_50,mouse IL-18_ of mFc-mIL-10, in this case, remained the same between the monotherapy and combination groups, as it reflected the interaction between mIL-10 and the mIL-10 alpha receptor. The EC_50,mouse IL-18_ was estimated to be 2.4 nM, similar to the *in vitro* binding affinity (K_d_ = 3.2 nM) of mFc-mIL-10 to the mIL-10 alpha receptor. Additionally, using the Bayesian estimation method, the turnover rate constant for mIL-18 was estimated to be 0.064 h^−1^, which yielded a turnover t_1/2_ of 11 h in mice.

#### 3.1.3 Correlation of Mouse IL-18 Induction With Antitumor Efficacy

**FIGURE 4:**
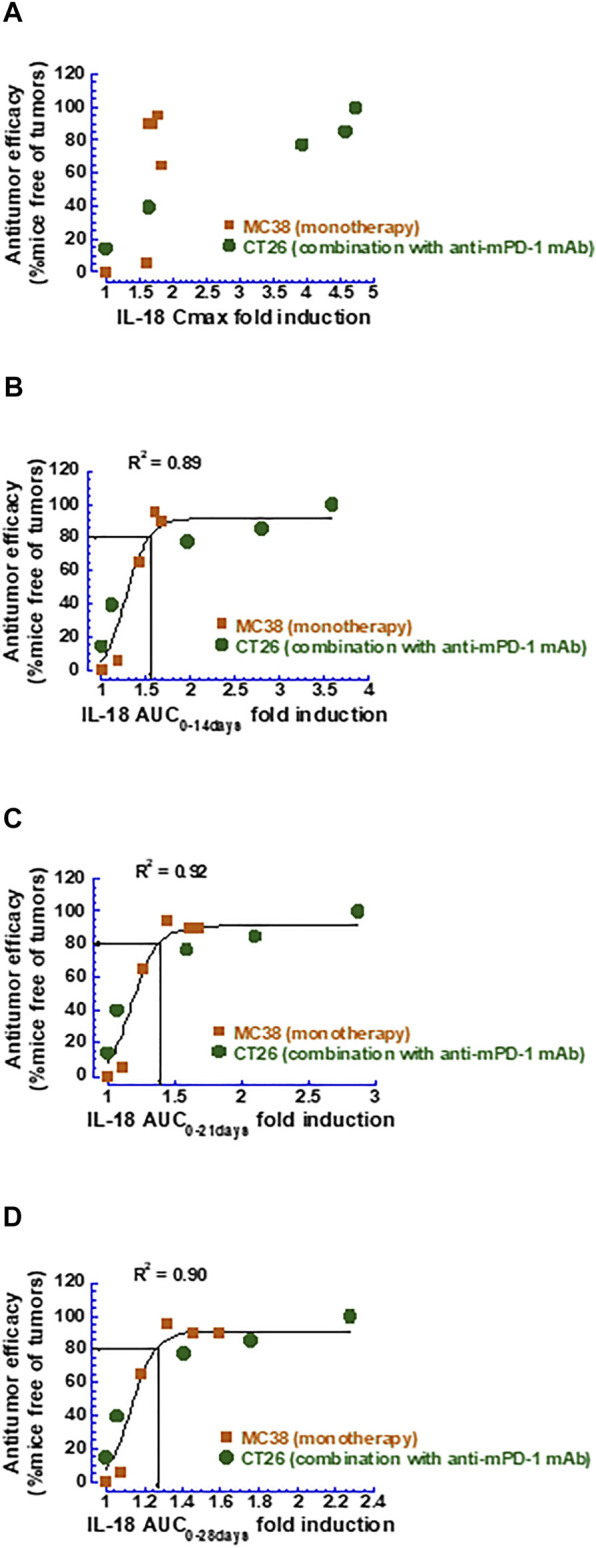
Correlation between various metrics of mouse IL-18 fold-induction and antitumor efficacy measured as the percentage of mice that were tumor-free. **(A)** Mouse IL-18 fold-induction based on C_max_; **(B)** Mouse IL-18 fold-induction based on AUC_0–14 days_; **(C)** Mouse IL-18 fold-induction based on AUC_0–21 days_; **(D)** Mouse IL-18 fold-induction based on AUC_0–28 days_.

Based on the PK/PD model established, the extent of the mIL-18 induction following the mFc-mIL-10 treatment as a single agent or in combination with the anti-mPD-1 mAb was simulated using the PK parameters listed in Table 1, except for the V_c_,_apparent_ (45 ml/kg) that was estimated from fitting the drug concentration data observed from efficacy studies in the MC38 and CT26 models (Yang et al., 2022). The simulated mIL-18 concentration-time profiles in these models are shown in Supplementary Figures S3A, S3B. The correlation of the mIL-18 fold-induction in the C_max_ or AUC was explored against the percentage of mice that were tumor-free in the MC38 and CT26 models (Figure 4). The result showed that the mIL-18 AUC fold-induction unified the efficacy readouts from two mouse syngeneic tumor models. Importantly, ≥80% of tumor-free mice can be achieved when the mIL-18 AUC fold-induction over 2, 3, and 4 weeks was ≥1.6, 1.4, and 1.3, respectively.

### 3.2 Cynomolgus Monkey Studies

#### 3.2.1 Pharmacokinetics and Antidrug Antibody Response After Intravenous Administration of Human IL-10 Human Fc-Fusion Protein to Cynomolgus Monkeys

**FIGURE 5:**
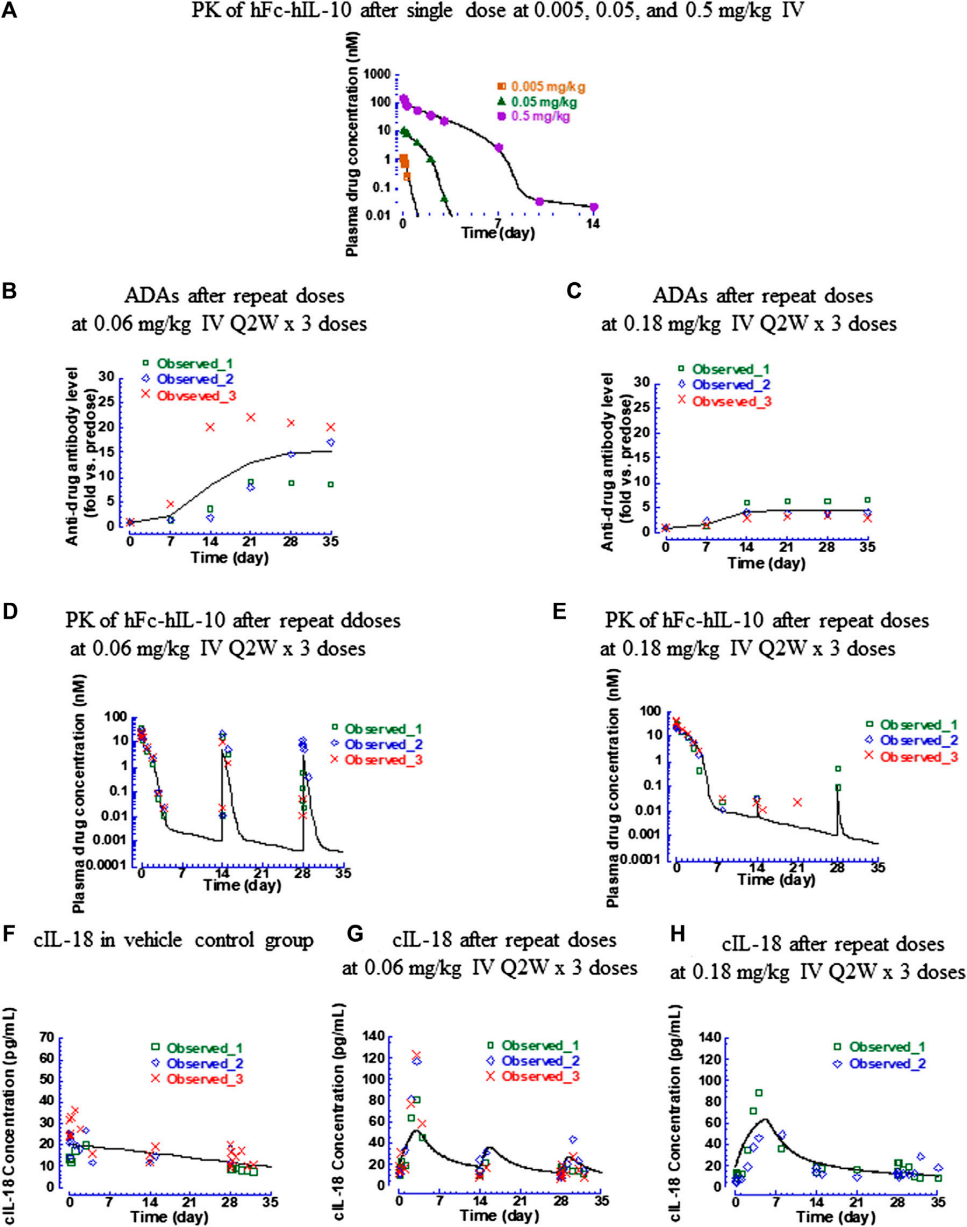
Pharmacokinetic, antidrug antibody, and IL-18 induction data of hFc-hIL-10 in cynomolgus monkeys. **(A)** Fitted versus observed pharmacokinetic data after single intravenous doses of 0.005, 0.05, and 0.5 mg/kg; **(B,C)** Time course of antidrug antibody levels after repeat intravenous doses (0.06 and 0.18 mg/kg Q2W x 3 doses); **(D,E)** Fitted versus observed pharmacokinetic data after repeat intravenous doses (0.06 and 0.18 mg/kg Q2W x 3 doses); **(F–H)** Fitted versus observed IL-18 induction data after repeat intravenous doses (0.06 and 0.18 mg/kg Q2W x 3 doses). At 0.18 mg/kg, the IL-18 data of one monkey were not included in the data analysis and not shown in the figure, because of a much higher predose baseline than the rest of the animals. Lines represent the model-fitted curves except for the antidrug antibody data where the line is the mean value of the group; symbols show individual observed data (*n* = 1 per time point for the single dose; *n* = 3 per time point for repeat doses). When data points are less than the number indicated, they are below the lower limit of quantitation for pharmacokinetic data or unavailable for cynomolgus monkey IL-18 data.

hFc-hIL-10 exhibited nonlinear PK in cynomolgus monkeys, similar to what was observed with mFc-mIL-10 in mice (Yang et al., 2022). After single IV doses of 0.005, 0.05, and 0.5 mg/kg (*n* = 1 per dose), the terminal t_1/2_ of hFc-hIL-10 was 0.11, 0.40, and 1 day, respectively (Figure 5A and Supplementary Table S3). The nonlinear PK of hFc-hIL-10 was attributed to TMDD, as demonstrated with mFc-mIL-10 (Yang et al., 2022). After repeat IV doses were given every 2 weeks for 3 doses at 0.06 and 0.18 mg/kg, significant ADA responses were observed (Figures 5B,C). The ADA effect on PK appeared to be dose-dependent (Figures 5D,E), although it was not reflected by the ADA titers due to the qualitative nature of the ADA assay where circulating drugs competed with the drug as a capturing agent in the assay.

The monkey hFc-hIL-10 PK data obtained from the single- and repeat-dose studies were fitted simultaneously by incorporating both target- and non-target-mediated elimination in the model, the same as what was conducted for the mouse data. The key models tested and model performance are summarized in Supplementary Table S4. The fitted versus observed PK data after a single dose and repeat administration are shown in Figures 5A,D,E, with the residual plot available in Supplementary Figure S4A. The estimated PK parameters are summarized in Table 2. To account for the drug exposure loss due to ADAs, a dose reduction factor was introduced to the model. For the 0.06-mg/kg dose, the dose reduction factor after the 2nd and 3rd dose was estimated to be 3 and 4.9, respectively; for the 0.18-mg/kg dose, the dose reduction factor after the 2nd and 3rd dose was 2,530 and 380, respectively. The K_m,target-mediated_ of hFc-hIL-10 was 0.44 nM, similar to the *in vitro* binding affinity K_d_ (0.3 nM) of hFc-hIL-10 to the IL-10 receptor alpha in cynomolgus monkeys. The results were comparable to those in mice, further supporting that TMDD is responsible for the nonlinear PK of hFc-hIL-10 in cynomolgus monkeys.

**TABLE 2:** Pharmacokinetic and pharmacodynamic parameters estimated for hFc-hIL-10 in cynomolgus monkeys after intravenous administration at 0.06- and 0.18-mg/kg doses given once every two weeks for three doses.

Parameter	Value estimated (mean ± standard error)
**Pharmacokinetics^a^**	
V_c_ (L/kg)	4.6 × 10^−2^ ± 5.1 × 10^−4^
k_12_ (1/h)	3.7 × 10^−2^ ± 5.6 × 10^−3^
k_21_ (1/h)	1.3 × 10^−1^ ± 1.9 × 10^−2^
k_13_ (1/h)	1.1 × 10^−3^ ± 1.3 × 10^−4^
k_31_ (1/h)	4.7 × 10^−3^ ± 1.9 × 10^−3^
k_el, non-target-mediated_ (1/h)	1.7 × 10^−2^ ± 6.5 × 10^−4^
K_m,target-mediated_ (nM)	4.4 × 10^−1^ ± 4.5 × 10^−2^
V_max,target-meidated_ (nmol/kg/h)	6.8 × 10^−3^ ± 2.5 × 10^−4^
**Pharmacodynamics (cIL-18 induction)**	
C_cIL-18,baseline,predose_ (pg/ml)	20 ± 0.95
Slope_cIL-18,baseline_ (pg/ml/h)	1.3 × 10^−2^ ± 2.3 × 10^−3^
E_max,cIL-18_ (pg/ml)	57 ± 12
EC_50,cIL-18_ (nM)	8.2 × 10^−2^ ± 1.2 × 10^−2^
k_out,cIL-18_ (1/h)	1.4 × 10^−2^ ± 4.8 × 10^−3^

a To account for the drug exposure loss as a result of antidrug antibodies, individual dose reduction factors were used. For the 0.06-mg/kg dose, the dose reduction factors after the 2nd and 3rd dose estimated from fitting the data were 3.0 ± 0.53 and 4.9 ± 2.8, respectively; for the 0.18-mg/kg dose, the corresponding dose reduction factors were 2,530 ± 714 and 380 ± 69, respectively. Please see Section 2.5.3 for the rationale and estimation methodology.

#### 3.2.2 Pharmacokinetic/Pharmacodynamic Modeling of Cynomolgus Monkey IL-18 Data After Repeat Doses of Human IL-10 Human Fc-Fusion Protein to Cynomolgus Monkeys

The induction of cIL-18 levels by hFc-hIL-10 was evaluated in a repeat-dose study at 0.06 and 0.18 mg/kg given every 2 weeks for 3 doses. Similar to the mIL-18 modeling work, an indirect response with drug stimulatory effects on the rate of cIL-18 production was used to describe the data, with the estimated PD parameters summarized in Table 2. The fitted versus observed cIL-18 data are shown in Figures 5F–H, and the residual plot is available in Supplementary Figure S4B. The key models tested and model performance is available in Supplementary Table S4.

Different from the mIL-18 data, there was no apparent time delay in the cIL-18 elevation. The estimated predose cIL-18 baseline level was 20 pg/ml, about 6 times lower than that in mice. The slope for describing the downward trend of cIL-18 levels in the vehicle control group was 0.013 pg/ml/hour. The time-dependent downward trend of cIL-18 in monkeys was opposite of the upward trend observed in mice. The rate of change was also slower in monkeys than in mice. One difference was that mice in the control group received an isotype control antibody, which could trigger an immune response causing the upward trend of the mIL-18 induction. In monkeys, only a vehicle was given in the control group, with no clear reason that could explain the decreases of cIL-18 over time.

The EC_50,cyno IL-18_ was estimated to be 0.082 nM, about 4-fold more potent than the *in vitro* binding affinity K_d_ (0.3 nM) of hFc-hIL-10 to the IL-10 receptor alpha in cynomolgus monkeys. The E_max,cyno IL-18_ was estimated to be 57 pg/ml versus 126 pg/ml estimated in mice. Additionally, the estimated k_out,cyno IL-18_ was 0.014 h^−1^, corresponding to the cIL-18 turnover t_1/2_ of 50 h in cynomolgus monkeys. The value is in line with the terminal t_1/2_ (26 h) measured with recombinant hIL-18 in cynomolgus monkeys (Herzyk et al., 2003).

#### 3.2.3 Pharmacokinetic/Pharmacodynamic Modeling of Thrombocytopenia and Anemia Data After Repeat Doses of Human IL-10 Human Fc-Fusion Protein to Cynomolgus Monkeys

**FIGURE 6:**
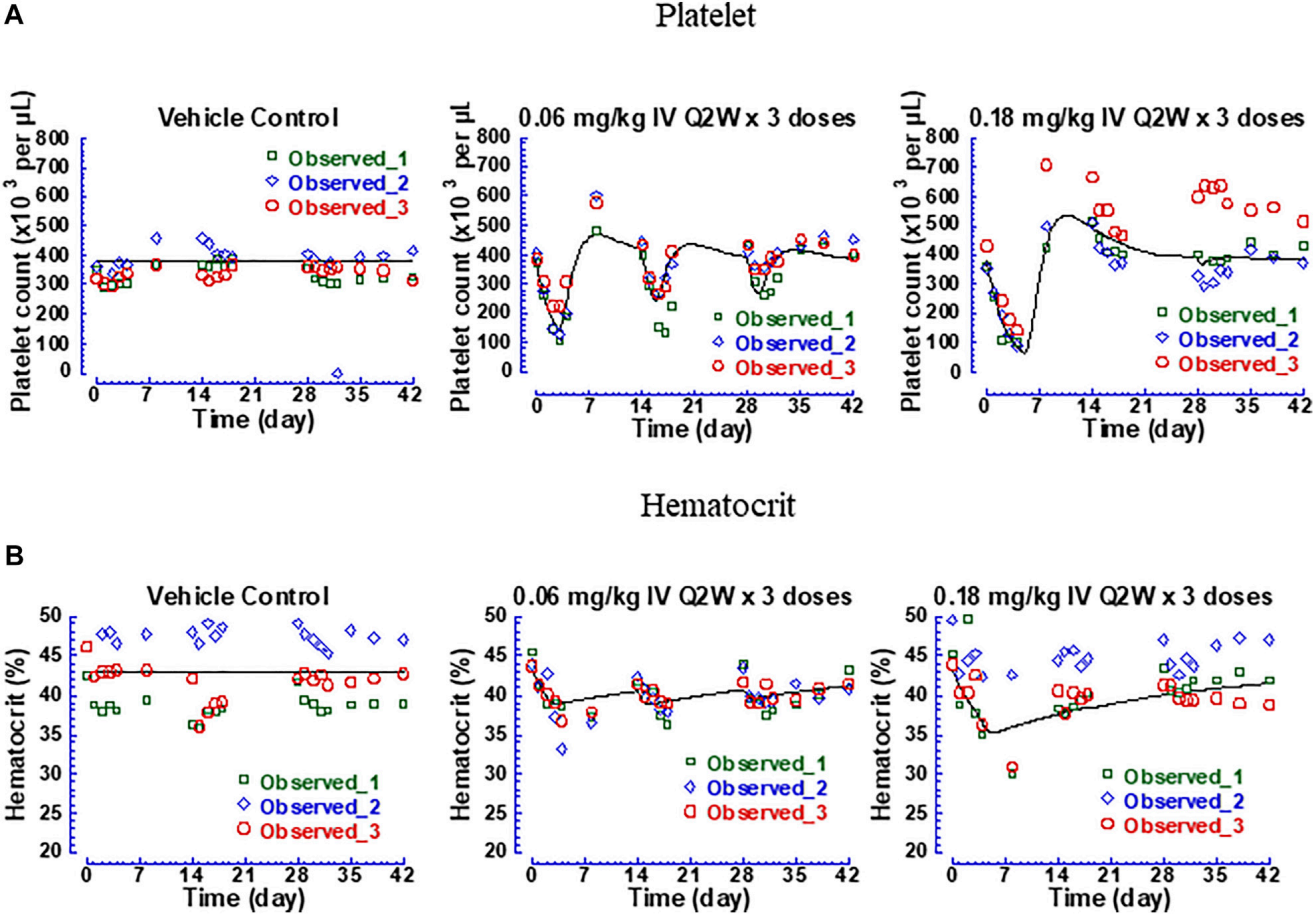
Fitted versus observed platelet count and hematocrit data of hFc-hIL-10 in cynomolgus monkeys after repeat intravenous doses of 0.06 and 0.18 mg/kg given once every 2 weeks for 3 doses. **(A)** Platelet counts; **(B)** Hematocrit. Lines represent the model-fitted curves; symbols show individual observed data (n = 3 per time point).

Thrombocytopenia and anemia were observed with hFc-hIL-10 in the same repeat-dose study where cIL-18 levels were measured. PK/PD models were developed to quantitatively evaluate the relationships between drug concentrations and hematological AEs. Figure 6 shows the fitted versus observed platelet count and hematocrit data at the doses of 0.06 and 0.18 mg/kg given every 2 weeks for 3 doses, with the residual plots shown in Supplementary Figures S5A, S5B and model performance available in Supplementary Table S4. The extent of decreases in platelet counts and hematocrit was dose- and drug concentration-dependent, with a loss in drug exposure leading to a less degree of hematological changes. The estimated IC_50_ of hFc-hIL-10 for thrombocytopenia and anemia was 0.034 and 0.58 nM, respectively (Table 3). The IC_50_ values for hematological AEs were in a concentration range comparable to the hFc-hIL-10 EC_50_ (0.11 nM) for the cIL-18 induction in cynomolgus monkeys.

**TABLE 3:** Pharmacodynamic parameters estimated for hematological changes in cynomolgus monkeys after intravenous administration of hFc-hIL-10 at 0.06- and 0.18-mg/kg doses given once every two weeks for three doses.

Parameter	Value estimated (mean ± standard error)
**Platelet count**	
C_cyno platelet,predose_ (x10^3^/µL)	379 ± 6.0
I_max,cyno platelet_ (%)	100% (fixed)
IC_50,cyno platelet_ (nM)	3.4 × 10^−2^ ± 1.4 × 10^−2^
k_out,cyno platelet_ (1/h)	1.6 × 10^−2^ ± 1.4 × 10^−3^
**Hematocrit**	
C_cyno hematocrit,predose_ (%)	43 ± 0.30
I_max,cyno hematocrit_ (%)	100% (fixed)
IC_50,cyno hematocrit_ (nM)	5.8 × 10^−1^ ± 3.3 × 10^−1^
k_out,cyno hematocrit_ (1/h)	1.9 × 10^−3^ ± 3.4 × 10^−4^

Additionally, the turnover rate constant for platelet counts and red blood cells was estimated to be 0.016 and 0.0019 h^−1^ (Table 3), respectively, corresponding to the turnover t_1/2_ of platelets and red blood cells of 1.8 and 16 days. The norm lifespan of platelets and red blood cells in rhesus monkeys was reported to be 6.5 and 85 days, respectively (Moroni et al., 2011). Assuming the same value as that in cynomolgus monkeys, the turnover t_1/2_ of platelets and red blood cells was estimated as the lifespan multiplied by 0.693, which yielded 4.5 and 58 days, respectively. Shorter turnover t_1/2_ values estimated from the PK/PD modeling work may reflect the trafficking of platelets and red blood cells in and out of the circulation as opposed to the true turnovers. Nevertheless, the values were in the same rank order as the lifespan data in monkeys.

### 3.3 Determination of Therapeutic Index

To further evaluate the safety risk associated with hematological changes, the efficacious dose of hFc-hIL-10 in cynomolgus monkeys was projected by targeting the same fold-induction of the cIL-18 AUC as what was observed in mice where robust antitumor efficacy was observed. Using the PK/PD model established for the cIL-18 induction in cynomolgus monkeys and assuming no ADA effect on drug exposure upon repeat dosing, the projected efficacious dose in cynomolgus monkeys was 0.025 mg/kg and was the same under different dosing regimens (Q2W, Q3W, or Q4W). The cIL-18 concentration-time profiles are shown in Supplementary Figure S6. At this dose, the cIL-18 AUC fold-induction over 2, 3, and 4 weeks was 1.6, 1.4, and 1.3, respectively, which corresponded to the mIL-18 AUC fold-induction that resulted in 80% tumor-free mice in efficacy evaluations.

A PK-based approach was also employed for the efficacious dose projection in cynomolgus monkeys by targeting the drug AUC_tot_ at the mouse efficacious doses and linearly correcting the exposure for a 10-fold difference in the *in vitro* receptor binding affinity between mice and cynomolgus monkeys (3.2 vs. 0.3 nM). Targeting the drug AUC_tot_ (25 nM*day) observed in mice at the 0.1-mg/kg single dose where robust efficacy (77% tumor-free mice) in the CT26 tumor model was observed in combination with the anti-mPD-1 mAb, the projected efficacious dose in cynomolgus monkeys after the affinity correction was 0.02 mg/kg. Applying the same approach and targeting the drug AUC_tot_ (100 nM*day) at the 0.3-mg/kg single dose where the monotherapy efficacy (65% tumor-free mice) was observed in the MC38 tumor model, the projected efficacious dose in cynomolgus monkeys was 0.05 mg/kg. The PK-based efficacious doses are aligned with the dose projected using the PD-based method.

**FIGURE 7:**
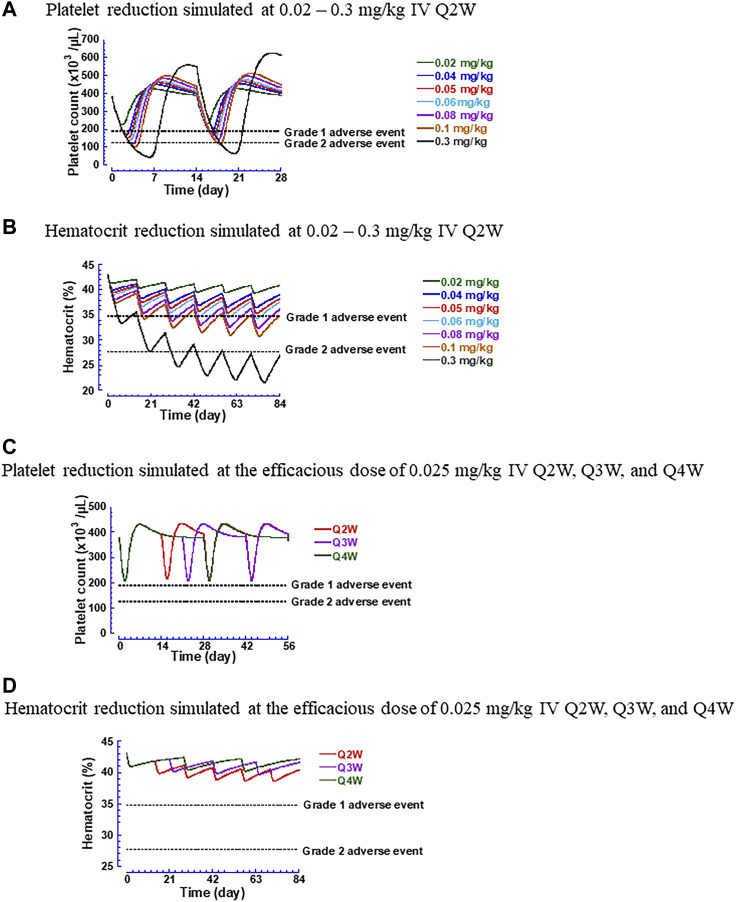
Simulated hematological changes produced by hFc-hIL-10 in cynomolgus monkeys. **(A)** Platelet counts at the intravenous doses of 0.02–0.3 mg/kg given once every 2 weeks; **(B)** Hematocrit at the intravenous doses of 0.02–0.3 mg/kg given once every 2 weeks; **(C)** Platelet counts at the efficacious dose of 0.025 mg/kg under intravenous dosing regimens of every 2, 3 and 4 weeks; **(D)** Hematocrit at the efficacious dose of 0.025 mg/kg under intravenous dosing regimens of every 2, 3 and 4 weeks. The Grades 1 and 2 adverse event lines are based on National Cancer Institute Common Terminology Criteria Adverse Events v5.0 and applied as the percentage of changes in hematological parameters to the baseline values estimated in cynomolgus monkeys.

Furthermore, simulations were performed to examine the time courses of thrombocytopenia and anemia after administering hFc-hIL-10 intravenously to cynomolgus monkeys. Figures 7A,B display the temporal profiles of platelet counts and hematocrit at a dose range of 0.02–0.3 mg/kg with a dosing frequency of every 2 weeks Figures 7C,D show the effect of dosing schedules on the changes in platelet counts and hematocrit at the monkey PD-based efficacious dose of 0.02 mg/kg administered under the Q2W, Q3W, and Q4W regimens. A less frequent dosing regimen helped to reduce the magnitude of red blood cell decreases but did not alter the extent of platelet count reduction. Based on the simulated results, the MTD in cynomolgus monkeys was determined. For thrombocytopenia, the dose producing a Grade 2 or less AEs was predicted to be 0.06 mg/kg and was independent of the dosing regimens. For anemia, the dose resulting in Grade 2 or less AEs was schedule-dependent; the predicted MTD at the dosing regimens of Q2W, Q3W, and Q4W was 0.14, 0.24, and 0.36 mg/kg, respectively.

Using the MTD and the efficacious dose projected in cynomolgus monkeys, the TI of hFc-hIL-10 can be determined in the same species. Table 4 summarizes the TI values in cynomolgus monkeys concerning thrombocytopenia and anemia. Thrombocytopenia appeared to be the dose-limiting toxicity. Using the PD-based efficacious dose, the TI was found to be x2.4. With the PK-based efficacious dose, the TI was x1.2–3. Collectively, these values indicate a narrow TI for hFc-hIL-10.

**TABLE 4:** Therapeutic index of hFc-hIL-10 in cynomolgus monkeys.

Method	Pharmacodynamic-based efficacious dose	Pharmacokinetic-based efficacious dose	
Efficacious dose (mg/kg)	0.025 mg/kg	0.020 mg/kg	0.050 mg/kg
	Q2W, Q3W, or Q4W	Q2W, Q3W, or Q4W for combination therapy with anti-PD-1 monoclonal antibody	Q2W, Q3W, or Q4W for monotherapy
Dose (mg/kg) producing Grade 2 or less thrombocytopenia		0.060 mg/kg Q2W, Q3W, or Q4W	
Dose (mg/kg) producing Grade 2 or less anemia		0.14 mg/kg Q2W	
		0.24 mg/kg Q3W	
		0.36 mg/kg Q4W	
Therapeutic index versus thrombocytopenia^a^	2.4x	3x	1.2x
Therapeutic index versus anemia^a^	5.6x (Q2W)	7x (Q2W)	2.8x (Q2W)
	9.6x (Q3W)	12x (Q3W)	4.8x (Q3W)
	14x (Q4W)	18x (Q4W)	7.2x (Q4W)

a The therapeutic index was calculated based on the ratio of the dose producing Grade 2 or less adverse events versus the efficacious dose.

Q2W, once every 2 weeks; Q3W, once every 3 weeks; Q4W, once every 4 weeks.

## 4 Discussion

In this work, PK/PD/efficacy relationships in mice, as well as PK/PD and PK/toxicity relationships in cynomolgus monkeys, were evaluated using model-based approaches. IL-18, as a PD endpoint, was demonstrated to be relevant to pharmacological effects and used to translate efficacy from mice to cynomolgus monkeys. An efficacious dose in monkeys was projected by targeting the fold-induction of the IL-18 AUC same as that observed in mice where a complete tumor regression was found in 80% of mice in efficacy evaluations. Additionally, a conventional PK-based approach was applied for the efficacious dose projection in monkeys by achieving the mouse efficacious exposure and correcting for the receptor binding affinity difference between the two species. Both PD- and PK-based methods resulted in comparable efficacious doses in cynomolgus monkeys and values were used as the basis for the TI determination. Furthermore, the MTD in cynomolgus monkeys was determined through PK/thrombocytopenia and PK/anemia relationships, allowing the TI assessment in the same species. The model-based approaches integrated the preclinical pharmacology and toxicology data and provided a high level of confidence in the TI in cynomolgus monkeys that exhibit toxicity relevant to human safety.

IL-18 was found to be a relevant PD biomarker that reflected the action of IL-10 Fc-fusion proteins. The elevation of IL-18 in both mice and cynomolgus monkeys was dependent on the dose and concentration of the Fc-fusion proteins. For example, when the drug exposure was diminished as a result of the ADA formation, the IL-18 induction also attenuated or disappeared after the 2nd or 3rd dose in the monkey repeat-dose study. The EC_50_ values corresponding to the IL-18 induction in mice and cynomolgus monkeys were also aligned with the *in vitro* binding affinity to the IL-10 receptor alpha, further connecting the IL-18 induction with the mechanism of action of IL-10 Fc-fusion proteins. Clinically, the fold of IL-18 induction produced by PEGylated IL-10 over the baseline was found to correlate best with objective tumor responses (Naing, et al., 2018). In our studies, the fold-induction of the IL-18 AUC correlated well with the antitumor efficacy in mouse syngeneic tumor models either as a monotherapy or in combination with an anti-mPD-1 mAb. Both preclinical and clinical data supported that the IL-18 induction was a functionally relevant biomarker associated with the IL-10 treatment for cancer and hence it was used as a common PD endpoint that connects mice to monkeys for the efficacious dose projection.

To translate the IL-18 induction response from mice to cynomolgus monkeys, the fold-induction in the IL-18 AUC, as opposed to the absolute value, was used as a basis for the efficacious dose projection. This is because the IL-18 baseline levels appeared to be different between mice and cynomolgus monkeys. The mIL-18 basal level estimated from PK/PD modeling was 113 pg/ml, which was 5.7-fold higher than the cIL-18 concentration (20 pg/ml) in cynomolgus monkeys. In humans, the baseline level of hIL-18 was reported to be 160–200 pg/ml (Koenig et al., 2006; Thompson et al., 2007). Reagents used in bioanalytical assays for IL-18 measurements could affect the results. Our cIL-18 assay was developed using human reagents. It is possible that the assay we had for cIL-18 only captured a portion of cIL-18 in cynomolgus monkeys. Hence, the fold-induction may be more appropriate for connecting the IL-18 induction between mice and cynomolgus monkeys. One additional complicating factor is that the IL-18 assays in our studies measured the total IL-18 level that included the active, unbound IL-18 and the bound complex with the IL-18 binding protein. In mice, the mIL-18 binding protein levels were found to be increased concordantly with mIL-18 concentrations upon the mFc-mIL-10 treatment, with a fold-increase in a magnitude similar to that of mIL-18 (data not shown). While the binding protein provided an independent indicator that mIL-18 was induced after the mFc-mIL-10 treatment, it is difficult to discern the downstream activity of mIL-18, because the binding protein has a high affinity to mIL-18 and hence reduces its unbound concentration for activity. Nevertheless, dose- and concentration-dependent total IL-18 increases were observed in both mice and cynomolgus monkeys following the treatment of IL-10 Fc-fusion proteins, suggesting that what was measured by the existing assays reasonably reflected the drug action of IL-10 Fc-fusion proteins.

In the preclinical setting, while mouse tumor models offer readouts on pharmacological activities, cynomolgus monkeys are often used as a model for human safety assessment. In this case, the cynomolgus monkey is a relevant and sensitive species to reflect the IL-10-related toxicity based on the preclinical and clinical experience with a recombinant hIL-10. Preclinically, the recombinant hIL-10 affected the hematopoietic system, leading to anemia and thrombocytopenia. These changes were observed only in cynomolgus monkeys, not in mice (Rosenblum et al., 2002). The no observable adverse effect level following 1 month of IV and subcutaneous (SC) administration to cynomolgus monkeys was determined to be 15 and 50 µg/kg/day, respectively. In contrast, no sign of toxicity was observed in mice at the dose level of 2 mg/kg/day for 3 months. Clinically, the recombinant hIL-10 was given to patients with Crohn’s disease once daily SC for 28 days (Fedorak et al., 2000; Schreiber et al., 2000), and anemia and thrombocytopenia were observed with mild to moderate severity (≤ Grade 2) at 10 and 20 µg/kg/day. Additionally, Grade 3 to 4 anemia or thrombocytopenia was observed with PEGylated hIL-10 in cancer patients when it was given daily SC at doses up to 40 µg/kg (Naing et al., 2016). These data indicate that the cynomolgus monkey is a relevant species for assessing the human safety risk of IL-10 Fc-fusion proteins.

Using cynomolgus monkeys as a toxicology model, the safety profile of hFc-hIL-10 was examined following IV administration of single and repeat doses. A learning-and-confirming paradigm was applied for building PK/PD models to describe the time course of anemia and thrombocytopenia data. Preliminary PK/PD models were developed to capture the limited hematological observations after single doses (data not shown). The models established were then used to inform the design of sample collections for measuring platelets and hematocrit in a monkey repeat-dose study. During the repeat-dose study, the PK/PD model was used to evaluate hematological changes. It was found that decreases in platelets and hematocrit were well predicted by the models after the 1st dose, but they were over-predicted after the 2nd and 3rd doses. It was therefore hypothesized that a significant drug exposure loss occurred after the 2nd and 3rd doses. The hypothesis was subsequently confirmed with the PK results, supporting the robustness of the PK/PD models. Also, drug concentration dependency and reversibility in hematological AEs of hFc-hIL-10 were consistent with the findings observed with the recombinant hIL-10 and PEGylated hIL-10.

From the PK/PD models, the IC_50_ values of hFc-hIL-10 for thrombocytopenia and anemia were estimated to be 0.034–0.58 nM. These values are in a concentration range comparable to the EC_50_ (0.13 nM) for the cIL-18 induction and the receptor binding affinity (0.3 nM). The mechanism of anemia and thrombocytopenia caused by hFc-hIL-10 is unknown. The induction of cIL-18 by hFc-hIL-10 could contribute to the added effect on anemia and thrombocytopenia, as a recombinant hIL-18 has been reported to cause anemia and thrombocytopenia in monkeys and humans (Herzyk et al., 2003; Robertson et al., 2006). Additionally, decreases in hematocrit and platelet counts in the circulation could be due to sequestration in peripheral tissues such as the spleen. One piece of evidence supporting this hypothesis was that the turnover t_1/2_ values of platelets and red blood cells estimated from PK/PD modeling were shorter than the turnover t_1/2_ estimated from the lifespan data, potentially reflecting the redistribution of platelets and red blood cells from peripheral tissues to the blood after the drug disappeared from the circulation.

To adequately evaluate the TI of hFc-hIL-10, it is preferable to evaluate it in the same species. Because toxicological profiles in cynomolgus monkeys reflect human safety, it was used as the species for the TI determination. To evaluate the TI in monkeys, monkey efficacious doses were projected using two methods. One was a conventional PK-based method, where the efficacious AUC observed at mouse efficacious doses was used and adjusted linearly for the difference in the receptor binding affinity between mice and monkeys. The other one was to use the PD endpoint (fold-induction of the IL-18 AUC) to translate efficacy from mice to monkeys. Although both approaches yielded comparable efficacious doses in monkeys, there are a few advantages of using the PD-based approach. First, PD response is a step closer than PK data to reflecting pharmacological effects. Second, the PD response takes into consideration of species differences in binding affinity and sensitivity in response, whereas a linear adjustment for the affinity difference adopted in the PK-based method may not be ideal, because a nonlinear relationship commonly exists between drug concentrations and responses (e.g., the E_max_ model). Therefore, a PD-based method for the efficacious dose projection is a preferred approach.

The TI is classically defined as the ratio of the dose that causes AEs to the dose that produces pharmacological effects (Muller and Milton, 2012), which is useful for medical practice. During drug discovery and development processes, however, exposure-centric approaches using the C_max_ or the AUC are more routinely used for assessing the TI. In our work, we adopted the dose-based approach for TI determination. With thrombocytopenia predicted to be dose-limiting toxicity for hFc-hIL-10, the dose-based TI using PD- and PK-based efficacious dose projection was 2.4 and 1.2–3, respectively. If the C_max_ is used as an exposure metric for the TI calculation, the exposure multiples are the same as the dose-based TI, because of a proportional relationship between the dose and the C_max_ for a biologic that has restricted distribution largely in the volume of plasma water. If the AUC is used for the TI estimation, the exposure multiples are higher than the dose-based TI, because hFc-hIL-10 exhibited nonlinear PK in cynomolgus monkeys. For the PD-based efficacious dose, the exposure multiples based on the AUC were calculated to be 3.8. For the PK-based efficacious doses, the AUC-based exposure multiples were 1.4 for the monotherapy and 5.5 for the combination therapy.

The limitation of the existing modeling work is that the average PK and PD data were used. Specifically, population PK/PD modeling work can be conducted with cynomolgus monkey data and enables the evaluation of variability among individual animals. The population-based approach is useful as the fold-induction is sensitive to the predose baseline value and provides more confidence in the population-based efficacious dose projection and the MTD determination. However, it should also be recognized that modeling average data affords speed and captures the central tendency of the data, which is sufficient to timely impact study designs and decision-making in drug discovery. One additional limitation is that extrapolations on the mIL-18 induction were made at doses higher than 0.3 mg/kg, for which we did not have actual experimental data to confirm. There is also uncertainty associated with the current TI determination. It is because mouse synergetic tumor models do not reflect heterogeneity in human cancer. Also, mouse tumors are implanted, which may trigger an immune response and are different from occurring human tumors. In these aspects, the TI value in humans may be smaller than that in cynomolgus monkeys, because the human efficacious dose needing to cover a broad patient population is potentially higher than the one currently projected.

In conclusion, the pharmacology and toxicology data of IL-10 Fc-fusion proteins were integrated quantitatively using model-based approaches. On the pharmacology front, through the modeling work, a PK/PD relationship was established in mice using the mIL-18 data as a functionally relevant PD biomarker that reflected the pharmacological action of mFc-mIL-10. The fold-induction of the mIL-18 AUC over the baseline was correlated quantitatively with the antitumor efficacy of mFc-mIL-10 in two mouse syngeneic tumor models. IL-18 as the PD endpoint then served as a bridge that connected mouse efficacy to that in cynomolgus monkeys using the cIL-18 induction PK/PD model developed for hFc-hIL-10. On the toxicology end, PK/PD models were successfully implemented for hFc-hIL-10 to describe the relationships between PK and hematological toxicity (anemia and thrombocytopenia) in the cynomolgus monkey that is predictive of the human safety risk based on the prior experience with a recombinant hIL-10. Using the PK/PD model, thrombocytopenia was predicted to be dose-limiting toxicity. With the efficacious dose and the MTD projected for hFc-hIL-10 in the cynomolgus monkey, the TI was then determined in the same species. Based on these results, hFc-hIL-10 was anticipated to have a narrow TI in the clinic, and the work was critical to the developability assessment of the molecule.

## Data Availability

The original contributions presented in the study are included in the article/Supplementary Materials, further inquiries can be directed to the corresponding author.
