# Supplementary material for: Application of Pharmacokinetic/Pharmacodynamic Modeling to Bridge Mouse Antitumor Efficacy and Monkey Toxicology Data for Determining the Therapeutic Index of an Interleukin-10 Fc Fusion Protein
Source: Front Pharmacol. 2022 Jun 20;13:829063. doi: 10.3389/fphar.2022.829063 (PMC9251408; doi:10.3389/fphar.2022.829063)

SUPPLEMENTARY MATERIAL

FRONTIERS IN PHARMACOLOGY

Manuscript title: Application of Pharmacokinetic/Pharmacodynamic Modeling to Bridge Mouse Antitumor Efficacy and Monkey Toxicology Data for Determining the Therapeutic Index of an Interleukin-10 Fc Fusion Protein

Manuscript ID: 829063

Authors: Zheng Yang, James Loy, Brian Poirson, Yanshan Dai, Surendran Rajendran, Shihua Xu, Vanessa Spires, Murali Gururajan, Zheng Lin, Jaren Arbanas, Stephen Carl, Samantha Pace, Yun Wang, John Mehl, Krishna Vasudevan, Thomas Spires, Ruslan Novosiadly, Shodeinde Coker, Raymond Perez, Kelly Covello, Paul Morin, Robert Graziano, Miranda Broz, Lois Lehman-Mckeeman

Affiliation: Bristol Myers Squibb, Princeton, New Jersey, U.S.A.

SUPPLEMENTARY METHODS

Bioanalytic Methods

1. Quantitation of mouse IL-10 mouse Fc-fusion protein

The concentrations of mouse IL-10 mouse Fc-fusion protein (mFc-mIL-10) in mouse samples (10% blood in Rexxip A buffer or serum) were measured using a microfluidic fluorescence immunoassay on a Gyrolab xP Workstation (Gyros AB, Uppsala, Sweden). A biotinylated rat anti-mIL-10 antibody (Southern Biotech, Birmingham, AL) was used as the capture reagent, and a goat anti-mouse immunoglobulin G (IgG) antibody labeled with Alexa Fluor 647 (Southern Biotech, Birmingham, AL) was used as the detection reagent. For diluted blood samples (10% blood in Rexxip A buffer), standard curves and quality control (QC) samples defining the dynamic range of the bioanalytical method were prepared in the 10% mouse blood with Rexxip A buffer and processed in the same fashion as the test samples. For serum samples, they were analyzed at 5-fold dilution in phosphate-buffered saline (PBS) with 1% bovine serum albumin (BSA) and 1M sodium chloride; the corresponding standard and QC samples were prepared in the same way as the serum test samples. Aliquots of diluted test samples, QC samples, standards, and reagents were added to 96-well polymerase chain reaction (PCR) microplates (Thermo Scientific, Cambridge, MA). The Gyrolab workstation transferred samples and reagents from the microplates to each of the microstructures within a Gyrolab Bioaffy compact disc (CD) and spun each CD at the optimized speed and time controlled by the Gyros software to ensure uniform optimal reaction times throughout the integrated assay workflow. All assay steps were automated and controlled by the Gyros control software. The mFc-mIL-10 concentrations in the test samples were quantified by a log-log linear-fit regression model using the SoftMax Pro software (Molecular Devices, Sunnyvale, California). Standard curves and QC samples were evaluated using target acceptance criteria for inaccuracy and imprecision of ± 20% of the nominal concentration to be considered acceptable for assay performance. The lower limit of quantitation (LLOQ) in diluted blood and

serum samples were 0.4 and 3 ng/mL (0.004 and 0.03 nM), respectively. The LLOQ in plasma after the conversion of drug concentrations from diluted blood samples using a theoretical dilution factor of 17.36 (see Section 2.5 Data Analysis for details) was 6.9 ng/mL (0.08 nM).

2. Quantitation of mouse IL-18

Serum mouse IL-18 (mIL-18) assay was developed using Simoa (single molecule array) homebrew 2.0 assay starter kit (Quanterix, Billerica, MA). The Simoa mIL-18 homebrew assay was a 3-step digital immunoassay using the Simoa HD-1 Analyzer. In the 3-step assay, mIL-18 antibody-coated paramagnetic beads were incubated with the samples. mIL-18 present in the samples was then captured by the mIL-18 antibody-coated beads. After washing, a biotinylated IL-18 detector antibody was incubated with the beads. The mIL-18 detector antibody was then bound to the captured mIL-18. Subsequently, streptavidin-β-galactosidase (SBG) was bound to the biotinylated mIL-18 detector antibody, resulting in the enzyme labeling of the captured mIL-18. Following a final wash, the beads were resuspended in a resorufin β-D-galactopyranoside (RGP) substrate solution and transferred to the Simoa Disc. Individual beads were then sealed within microwells in the array. The β-galactosidase hydrolyzed the RGP substrate in the microwell into a fluorescent product that provided the signal for the quantification.

Coupling of mIL-18 capture antibody to the Quanterix magnetic beads utilized carboxyl groups on the beads to conjugate to primary amines on the antibody via a 1-ethyl-3-(-3-dimethyl aminopropyl)carbodiimide hydrochloride (EDC) linker. Beads (200 µL at 1.4 x $10E^9$ /mL) were activated by EDC (0.5 mg/mL) for 30 minutes at 4°C. The capture mIL-18 antibody (200 µL at 0.2 mg/mL in the conjugation buffer) was added to the activated beads and incubated for 3 hours at 4°C. The mIL-18 antibody-conjugated beads were washed and blocked with BSA. To prepare for test samples, an aliquot of 5 µL serum sample was diluted into 145 µL of the sample diluent at 30-fold dilutions. The mIL-18 calibration curve was prepared from the mIL-18 ELISA kit (MBL International Corporation, Woburn, MA), in which a stock solution of 2500 pg/mL was made in calibration diluent (from Simoa homebrew kit). The standards ranged from 0 to 250 pg/mL.

The mIL-18 assay was run on the Simoa HD-1 Analyzer (Quanterix, Billerica, MA) as a 3-step run. The Simoa mouse IL-18 assay reagents were loaded into the reagent bay in the HD-1 analyzer: 5 mL of bead reagent, 12 mL of detector reagent containing the biotinylated IL-18 antibody at 1:1000 dilution, and 12 mL of SBG Reagent (150 pM) per 96-sample run. The samples, calibrators, and RGP (resorufin-β-D-galactopyranoside) were loaded into the sample bay. The Simoa mIL-18 assays used a 4-parameter curve fit data reduction method ($1/y^2$ weighted) to generate the calibration curve. The LLOQ of the assay was 0.11 pg/mL. After correcting for sample dilution factors, the LLOQ in serum samples was 3.3 pg/mL.

3. Quantitation of human IL-10 human Fc-fusion protein

The concentrations of human IL-10 human Fc-fusion protein (hFc-hIL10) in the cynomolgus monkey plasma samples were measured using a chemiluminescence immunoassay platform. Commercial rat anti-hIL-10 (Southern Biotech, Birmingham, AL) was used as the capture reagent. Samples, standards, and QCs were brought up to a final matrix concentration of 10% monkey plasma in PBS with 1% BSA and 0.05% Tween 20 (assay buffer) and loaded in the capture reagent coated, blocked 96-well flat-bottom Nunc MaxiSorp black plate (Thermo Fisher Scientific,

Waltham, MA). After overnight incubation at 4°C and wash steps, the detection reagent, biotin-labeled mouse anti-human IgG Fc mAb was added. Followed by another incubation for 2 hours at 4°C and wash steps, NeutrAvidin protein conjugated with horseradish peroxidase (Thermo Fisher Scientific, Waltham, MA) was added. After final incubation and wash steps, Pico substrate solution (Thermo Fisher Scientific, Waltham, MA) was added to assay plates and read in SpectraMax plate reader at the luminescence mode. The concentrations of hFc-hIL-10 in monkey plasma samples were calculated from luminescence intensity using a log-log linear calibration curve. Calibrators and QCs prepared in monkey plasma were diluted 10-fold in assay buffer and analyzed on each assay plate along with samples to ensure acceptable assay performance. Assay performance was within the acceptable range: % CV of the standards and QC was below 20 %, and QC recovery was within ± 20 % of the nominal values. The LLOQ in monkey plasma samples was 0.5 ng/mL (0.0055 nM).

4. Quantitation of cynomolgus monkey IL-18

A human IL-18 (hIL-18) Simoa kit from Quanterix (Billerica, MA) was evaluated for determining the concentrations of cynomolgus monkey IL-18 (cIL-18), due to a high sequence homology between the two species. The kit cross-reacted with the cIL-18 protein standard (ProcartaPlex NHP IL-18 simplex kit, ThermoFisher, Waltham, MA). The average recovery relative to hIL-18 (i.e., cIL-18 ÷ hIL-18 x100%) at three concentrations (0.375, 3.75, and 37.5 pg/mL) was 101.5%.

The Simoa human IL-18 assay kit is a 3-step digital immunoassay using the Simoa HD-1 Analyzer. The 3-step assay principle was the same as that described in the mIL-18 Simoa assay. The cIL-18 standards were prepared from the hIL-18 kit using a stock solution of 1000 pg/mL in the calibration diluent. The standard concentration ranges from 0 to 45 pg/mL. The plasma samples (3 µL) were diluted into 150 µL of the sample diluent and run on the Simoa HD-1 Analyzer using a 3-step run protocol. The Simoa cIL-18 assay uses a 4-parameter curve fit data reduction method ($1/y^2$ weighted) to generate the calibration curve. The LLOQ of the assay was 0.011 pg/mL. After accounting for the sample dilution, the LLOQ in plasma samples was determined to be 0.56 pg/mL.

5. Semi-quantitative detection of antidrug antibody in cynomolgus monkeys

Antidrug antibodies (ADA) in monkey plasma samples were detected on a chemiluminescence platform. hFc-hIL-10 and a mouse anti-monkey IgG with horseradish peroxidase (Southern Biotech, Birmingham, AL) were used as the capture and detection reagents respectively in 96-well flat-bottom Nunc MaxiSorp black plate (Thermo Fisher Scientific, Waltham, MA). Samples were analyzed at a 10-fold dilution in assay buffer (PBS with 1% BSA and 0.05% Tween 20) and incubated in the capture reagent coated, blocked assay plate overnight at 4°C. After incubation and wash steps the detection reagent was added. After another incubation for 2 hours at 4°C followed by wash steps, Pico substrate solution (Thermo Fisher Scientific, Waltham, MA) was added and read in SpectraMax plate reader at luminescence mode. The presence of detectable anti-hFc-hIL-10 antibodies in monkey plasma samples was determined by comparing the sample raw signal to the pre-dose raw signal. The samples with a signal higher than the pre-dose signal were considered positive.

6. Hematology

Peripheral blood smears from the sample collected were prepared and analyzed using the ADVIA 2120i Hematology System (Siemens Healthineers AG, Erlangen, Germany).

SUPPLEMENTARY TABLE

Supplementary Table S1. Summary of Study Designs and Experimental Data

	Study Design	Experimental Data
**Mouse antitumor efficacy studies with mFc-mIL-10**		
1) MC38 syngeneic tumor model in female C57BL6 mice (monotherapy)		
Study#1	Single IP dose<br>at 0.1, 0.3, 1, 3, and 10 mg/kg	Efficacy data.
Study#2	Single IP dose<br>at 0.1, 0.3, 1, 3, and 10 mg/kg	Efficacy data;<br>**mFc-mIL-10 concentration**: blood (60 µL) was collected serially via submandibular bleeds to obtain serum samples at 4 and 168 hours from tumor-bearing mice in the efficacy study at all dose levels (N = 5 per time point); additionally, blood (60 µL each) was harvested via submandibular bleeds to collect serum samples compositely at 1, 5, 24, 72, 168, 240, and 336 hours compositely from the satellite groups of non-tumor-bearing mice at 0.3 and 3 mg/kg in the same study (N = 3 per time point). All the study samples were stored at ≤-70°C until sample analysis for mFc-mIL-10 concentrations.
2) CT26 syngeneic tumor model in female BALB/c mice (combination with anti-mPD-1 antibody given 10 mg/kg IP Q4D for 3 doses)		
Study#1	Single IP dose<br>at 0.1, 0.3, and 1 mg/kg	Efficacy data;<br>**mFc-mIL-10 concentration**: blood (10 µL) was collected compositely via microsampling on the tail at 4, 48, 96, 168, 336, and 504 hours from tumor-bearing mice in the efficacy study at all dose levels (N = 4 or 6 per time point) and diluted into 90 µL Rexxip buffer (Gyros AB, Uppsala, Sweden) for analyses. All the study samples were stored at ≤-70°C until sample analysis for mFc-mIL-10 concentrations.
Study#2	Single IP dose<br>at 0.1, 0.3, and 1 mg/kg	Efficacy data;<br>**mFc-mIL-10 concentration**: blood (10 µL) was harvested serially via microsampling on the tail at 4, 24, 48, 96, and 168 hours from tumor-bearing mice in the efficacy study at all dose levels (N = 4 per time point) and diluted into 90 µL Rexxip buffer for analyses. All the study samples were stored at ≤-70°C until sample analysis for mFc-mIL-10 concentrations.

	Study Design	Experimental Data
Study#3	Single IP dose<br>at 0.03, 0.1, and 0.3 mg/kg	Efficacy data;<br>**mFc-mIL-10 concentration**: blood (10 µL) was obtained serially via microsampling on the tail at 4, 24, 48, 96, 168, and 192 (or 216) hours from tumor-bearing mice in the efficacy study at all dose levels (N = 4 per time point) and diluted into 90 µL Rexxip buffer for analyses. All the study samples were stored at ≤-70°C until sample analysis for mFc-mIL-10 concentrations.
**Mouse PK/PD study on mIL-18 induction in CT26-tumor-bearing mice (female BALB/c) treated with mFc-mIL-10**	Single IP dose<br>at 0.1 and 0.3 mg/kg (monotherapy)<br>at 0.1 and 0.3 mg/kg (combination therapy with anti-mPD-1 antibody given at 10 mg/kg every 4 days for 3 doses)	**mFc-mIL-10 concentration**: blood (10 µL) was collected serially via microsampling on the tail at 4, 48, and 96 hours from tumor-bearing mice in the study (N = 4 per time point) and diluted into 90 µL Rexxip buffer for analyses.<br>**mIL-18 concentration**: blood (30 µL) was harvested compositely via submandibular bleeds to obtain serum samples at 0, 72, 168, 240, 336, 384, and 432 hours from tumor-bearing mice in the study (N = 4 per time point).<br>All the study samples were stored at ≤-70°C until sample analysis for mFc-mIL-10 concentrations.
**Cynomolgus monkey single-dose study with hFc-hIL-10**	Single IV dose<br>0.005, 0.05, and 0.5 mg/kg (N = 1 male per dose)	**hFc-hIL-10 concentration**: blood (0.5 mL) was collected serially from the femoral vein into K_2EDTA tubes and centrifuged at 4°C (1500 – 2000 x g) to obtain plasma samples at predose, 0.25, 2, 6, 24, 48, 72, 168, 240, and 336 hours after the dose. All the study samples were stored at ≤-70°C until sample analysis for hFc-hIL-10 concentrations.
**Cynomolgus monkey repeat-dose study with hFc-hIL-10**	Repeat IV dose administered every 2 weeks for 3 doses<br>at 0.06 mg/kg (N = 1 male and 2 females) and<br>at 0.18 mg/kg (N = 2 males and 1 female)	**hFc-hIL-10 concentration**: blood (0.5 mL) was harvested serially from the femoral vein into K_2EDTA tubes and centrifuged at 4°C (1500 – 2000 x g) to obtain plasma samples at predose, 0.17, 1, 3, 7, 24, 48, 72, 96, and 168 hours (0.18 mg/kg only) post the 1st and 3rd doses; and at predose, 1, 24, and 168 hours (0.18 mg/kg only) post the 2nd dose. Samples were stored at -70°C until analysis for determining hFc-hIL-10 concentrations.<br>**cIL-18 concentration**: same samples collected for hFc-hIL-10 concentrations were used. Samples were stored at -70°C until analysis for determining cIL-18 levels.<br>**Hematological (hematocrit and platelet) assessment:** blood (1 mL) was collected serially from the femoral vein into K_2EDTA tubes at predose, 24, 48, 72, 96, and 192 hours after the 1st dose; at predose, 24, 48, 72, and 96 hours after the 2nd dose; and at

	Study Design	Experimental Data
		predose, 24, 48, 72, 96, 168, 240, and 336 hours after the 3rd dose. The samples were freshly analyzed.<br><br>**Anti-hFc-hIL-10 antibodies:** blood (0.2 mL) was harvested serially from the femoral vein to obtain serum samples at predose and subsequently every week until the 6th week after the initial dosing. Samples were stored at -70°C until analysis for determining hFc-hIL-10 concentrations.

Note: Submandibular bleeds were performed with the use of a sterile 5mm Goldenrod animal lancet (Braintree Scientific Inc., Braintree, MA). Animals were briefly restrained and an applicable volume of blood was collected from a puncture made proximal of the mandibular bone where the submandibular vein and facial vein converge.

Supplementary Table S2. Key Models Tested and Fitting Performance for Pharmacokinetic/Pharmacodynamic Modeling of mIL-18 Induction Data after Intraperitoneal Administration of mFc-mIL-10 to Mice

	Objective function[a,b]	Akaike information criterion[b]	Schwarz-Bayesian information criterion[b]
**PK model**			
1-compartment model with **one** $V_{c,apparent}$ values to be estimated	176	185	188
1-compartment model with **two** $V_{c,apparent}$ values to be estimated	165	177	180
2-compartment model with **one** $V_{c,apparent}$ value to be estimated	152	162	165
2-compartment model with **two** $V_{c,apparent}$ values to be estimated (**final model**)	134	146	149
**mIL-18 induction**			
4 transit compartments[c], time-dependent change **same** for both isotype and anti-PD-1 control groups, **different** Emax values between mono- and combo-therapy	375	399	419
5 transit compartments[c], time-dependent change **same** for both isotype and anti-PD-1 control groups, **different** Emax values between mono- and combo-therapy	373	396	416
6 transit compartments[c], time-dependent change **same** for both isotype and anti-PD-1 control groups, **different** Emax values between mono- and combo-therapy	370	394	414
Indirect response model with 72-hour **point delay** in drug administration, time-dependent change **same** for both isotype and anti-PD-1 control groups, **same** Emax value between mono- and combo-therapy	379	401	419
Indirect response model with 72-hour **point delay** in drug administration, time-dependent change **same** for both isotype and anti-PD-1 control groups, **different** Emax values between mono- and combo-therapy	332	356	376
Indirect response model with 72-hour **point delay** in drug administration, time-dependent change **different** for isotype and anti-PD-1 control groups, **different** Emax values between mono- and combo-therapy (**final model**)	328	353	375

a. Expressed as -2 times the log-likelihood function.

b. Converted from the output statistics in SAAM II.

c. The transit compartments herein refer to the compartments before the last compartment where the response was measured and modeled.

Supplementary Table S3. Noncompartmental Analysis of hFc-hIL-10 Pharmacokinetic Data after Intravenous Administration to Cynomolgus Monkeys at Single Doses of 0.005, 0.05, and 0.5 mg/kg

**Dose (mg/kg)**	**0.005**	**0.05**	**0.5**
C_{max} (nM)	1.16	12	149
T_{max} (h)	0.25	0.25	0.25
AUC_{tot} (nM*d)	0.20	10	198
CL_{tot} (mL/d/kg)	278	57	28
V_{ss} (mL/kg)	45	40	51
Terminal $t_{1/2}$ (d)	0.11	0.40	1.0

Note: Noncompartmental analysis was conducted using PKSolver (Zhang, Y., Huo, M., Zhou, J., Xie, S. (2010) PKSolver: An add-in program for pharmacokinetic and pharmacodynamic data analysis in Microsoft Excel. Comput. Methods Programs Biomed. 99:306-314. doi: 10.1016/j.cmpb.2010.01.007)

Supplementary Table S4. Key Models Tested and Fitting Performance for Pharmacokinetic/Pharmacodynamic Modeling of cIL-18 Induction, Thrombocytopenia, and Anemia Data after Intravenous Administration of hFc-hIL-10 to Cynomolgus Monkeys

	**Objective function[a,b]**	**Akaike information criterion[b]**	**Schwarz-Bayesian information criterion[b]**
**PK model**			
2-compartment model with Bayesian-estimated dose reduction factors	678	706	733
3-compartment model with fixed values of dose reduction factors based on fold-reduction in C_{max}	717	742	766
3-compartment model with Bayesian-estimated dose reduction factors (**final model**)	654	686	716
**cIL-18 induction**			
**No reduction** in the E_{max} of cIL-18 induction and constant baseline in the vehicle control group	443	456	471
**Reduction** in the E_{max} of cIL-18 induction at 2nd and 3rd doses and constant baseline in the vehicle control group	442	457	473
**Reduction** in the E_{max} of cIL-18 induction at 2nd and 3rd doses and a time-dependent baseline in the vehicle control group	428	445	464
**No reduction** in the E_{max} of cIL-18 induction and a time-dependent baseline in the vehicle control group (**final model**)	429	444	461
**Thrombocytopenia**			
Indirect response model with an estimated predose baseline (inhibition of production)	623	635	648
Indirect response model with a fixed predose baseline based on the median value from the vehicle control group and with a **precursor** (inhibition of production)	95	605	616
Indirect response model with an estimated predose baseline and with a **precursor** (inhibition of production) (**final model**)	89	602	614
**Anemia**			
Indirect response model with a fixed predose baseline based on the median value from the vehicle control group (inhibition of production)	230	239	250
Indirect response model with an estimated predose baseline (inhibition of production) (**final model**)	224	236	248

a. Expressed as -2 times the log-likelihood function.

b. Converted from the output statistics in SAAM II.

Supplementary Table S5. List of Abbreviations and Acronyms

Term	Definition
ADA	Anti-drug antibodies
AE	Adverse events
A_{IP}	Amount of drug at the absorption site after intraperitoneal administration
$A_{peripheral}$	Amount of drug in the peripheral compartment
C_{cIL-18}	Cynomolgus monkey IL-18 concentration
$C_{cIL-18,baseline,predose}$	Cynomolgus monkey IL-18 concentration before drug treatment
$C_{cyno\ hematocrit}$	Hematocrit in cynomolgus monkeys
$C_{cyno\ hematocrit,predose}$	Hematocrit in cynomolgus monkeys before drug treatment
$C_{cyno\ platelet}$	Cynomolgus monkey platelet concentration
$C_{cyno\ platelet\ precursor}$	Cynomolgus monkey platelet precursor concentration
$C_{cyno\ platelet\ precursor,predose}$	Cynomolgus monkey platelet precursor concentration before drug treatment
$C_{cyno\ platelet,predose}$	Cynomolgus monkey platelet concentration before drug treatment
cIL-18	Cynomolgus monkey interleukin-18
C_{mIL-18}	Mouse IL-18 concentration
$C_{mIL-18,baseline,predose}$	Mouse IL-18 concentration before drug treatment
C_p	Drug concentration in the central compartment
Cyno	Cynomolgus monkey
$EC_{50,cIL-18}$	Drug effective concentration corresponding to the half of the maximum IL-18 increases in cynomolgus monkeys
$EC_{50,mIL-18}$	Drug concentration corresponding to the half of the maximum IL-18 increases in mice
$E_{max,cIL-18}$	Maximum IL-18 increases in cynomolgus monkeys
$E_{max,mIL-18}$	Maximum IL-18 increases in mice
Fc	Fragment crystallizable
hFc-hIL-10	Human fragment crystallizable fused with human interleukin-10
hIL-10	Human interleukin-10
$IC_{50,cyno\ hematocrit}$	Drug inhibitory concentration corresponding to the half of the maximum hematocrit reduction in cynomolgus monkeys
$IC_{50,cyno\ platelet}$	Drug inhibitory concentration corresponding to the half of the maximum platelet reduction in cynomolgus monkeys
IgG	Immunoglobin G
$I_{max,cyno\ hematocrit}$	Maximum hematocrit reduction in cynomolgus monkeys
$I_{max,cyno\ platelet}$	Maximum platelet reduction in cynomolgus monkeys
k_{12}	Transfer rate constant from the central to the peripheral compartment
k_{13}	Transfer rate constant from the central to the 2nd peripheral compartment
k_{21}	Transfer rate constant from the peripheral to the central compartment
k_{31}	Transfer rate constant from the 2nd peripheral to the central compartment
k_a	Absorption rate constant
K_d	Dissociation constant for receptor binding affinity

Term	Definition
$k_{el,non\text{-}target\text{-}mediated}$	Non-target-mediated first-order elimination rate constant
$K_{m,target\text{-}mediated}$	Binding affinity to target
$k_{out,cIL\text{-}18}$	Turnover rate constant of cynomolgus monkey IL-18
$k_{out,cyno\ hematocrit}$	Turnover rate constant of hematocrit in cynomolgus monkeys
$k_{out,cyno\ platelet}$	Turnover rate constant of platelets in cynomolgus monkeys
$k_{out,mIL\text{-}18}$	Turnover rate constant of mouse IL-18
LLOQ	Lower limit of quantitation
mAb	Monoclonal antibody
mFc-mIL-10	Mouse fragment crystallizable fused with mouse interleukin-10
mIL-10	Mouse interleukin-10
mIL-18	Mouse interleukin-18
mPD-1	Mouse programmed death-1 receptor
MTD	Maximum tolerated dose
PBS	Phosphate-buffered saline
Pharmacodynamic(s)	PD
Pharmacokinetic(s)	PK
Q2W	Once every two weeks
Q3W	Once every three weeks
Q4W	Once every four weeks
$slope_{cIL\text{-}18,baseline}$	Rate of cynomolgus monkey IL-18 change in a control group over time
$slope_{mIL\text{-}18,baseline}$	Rate of mouse IL-18 change in a control group over time
TI	Therapeutic index
TMDD	Target-mediated drug disposition
V_c	Volume of distribution in the central compartment
$V_{c,apparent}$	Apparent volume of distribution in the central compartment after intraperitoneal administration
$V_{max,target\text{-}mediated}$	Maximum elimination rate mediated by target

SUPPLEMENTARY FIGURE

Supplementary Figure S1. Representative simulated vs. observed mFc-mIL-10 concentration-time profiles in efficacy studies following single-dose intraperitoneal administration at a range of doses

A. MC38 model

mFc-mIL-10 concentrations in the MC38 model after single-dose IP administration
(symbols were observed data from animals in the efficacy study; mean ± SD, N = 5)

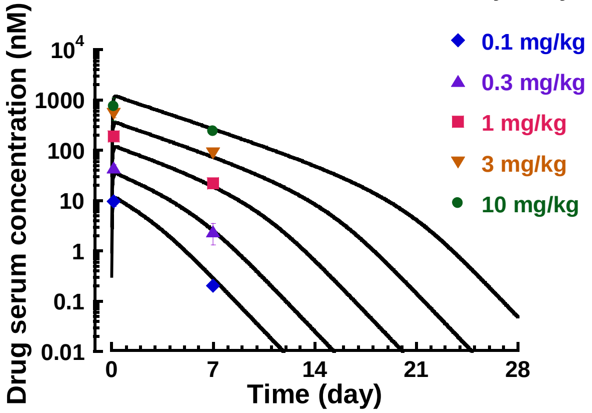

B. CT-26 model

mFc-mIL-10 concentrations in the CT26 model after single-dose IP administration
(symbols were observed data from animals in the efficacy study; mean ± SD, N = 4-6)

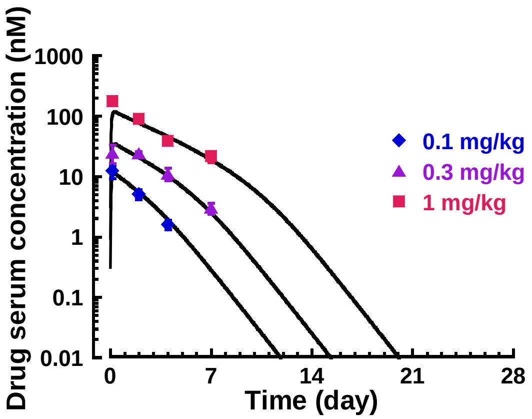

Supplementary Figure S2. Residual plots obtained from pharmacokinetic/pharmacodynamic modeling of average PK and mIL-18 induction data in mice

A. PK data

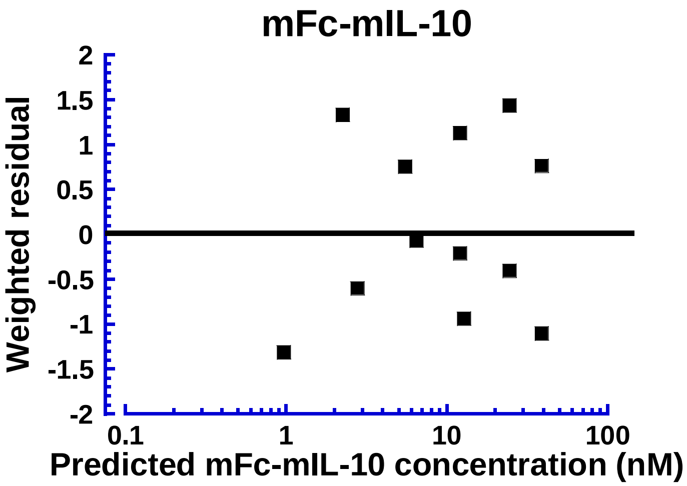

B. PD data

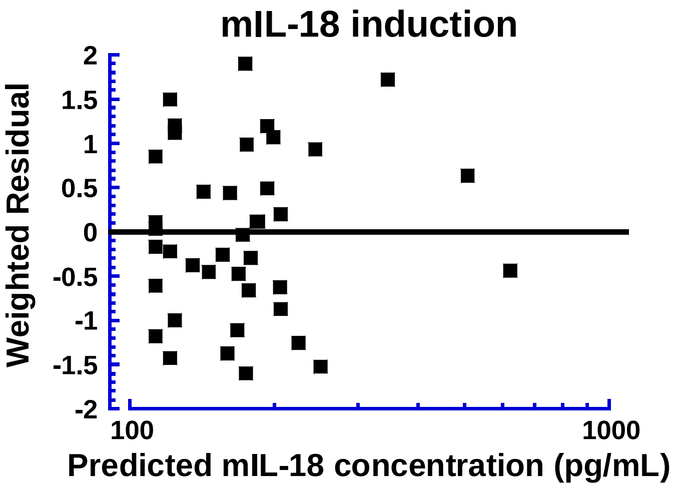

Supplementary Figure S3. Simulated mIL-18 concentration-time profiles in mouse syngeneic tumor models following single-dose intraperitoneal administration of mFc-mIL-10 at a range of doses

A.

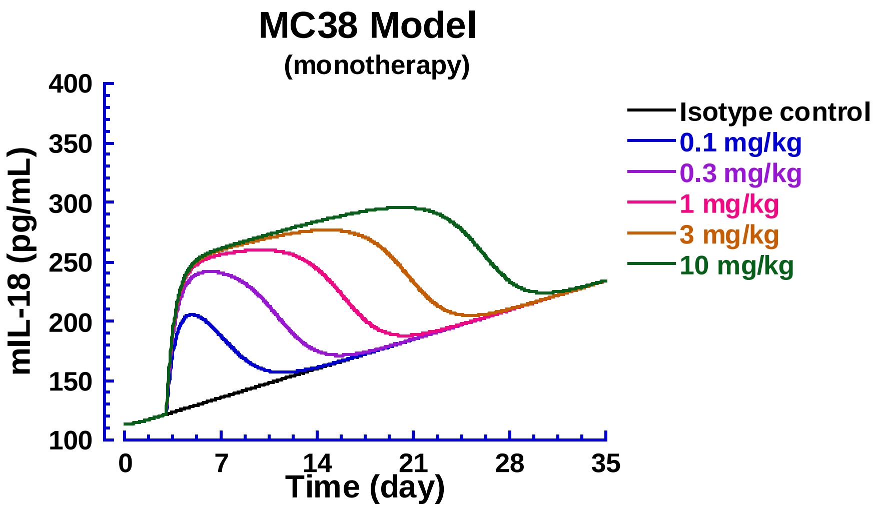

B.

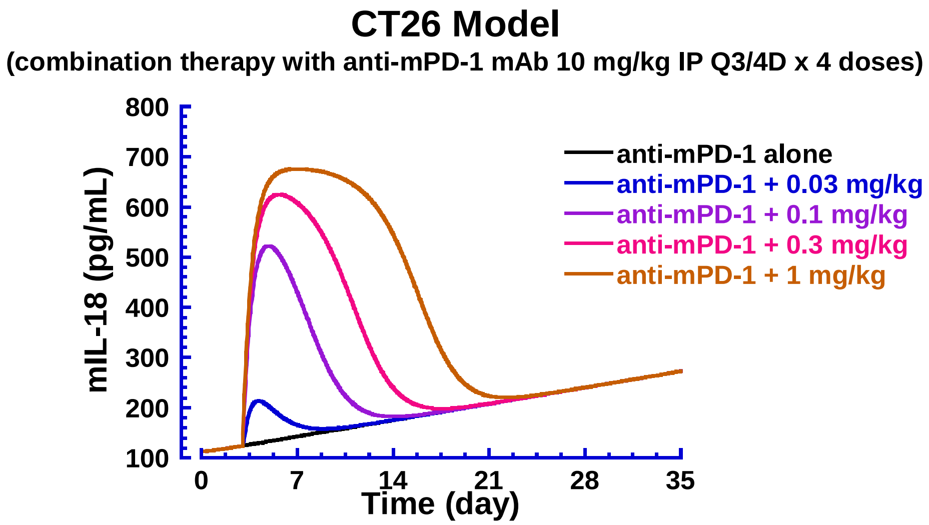

Supplementary Figure S4. Residual plots obtained from pharmacokinetic/pharmacodynamic modeling of average PK and cIL-18 induction data in cynomolgus monkeys

A. PK data
(single-dose IV data at 0.005, 0.05, and 0.5 mg/kg;
repeat-dose IV data at 0.06 and 0.18 mg/kg Q2W x 3 doses)

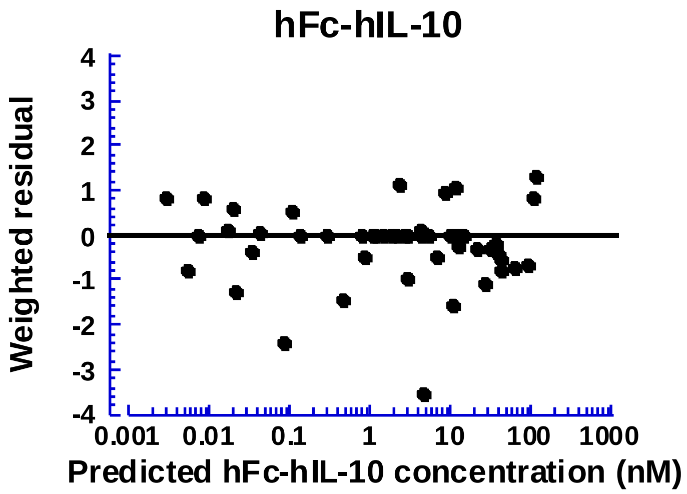

B. PD data

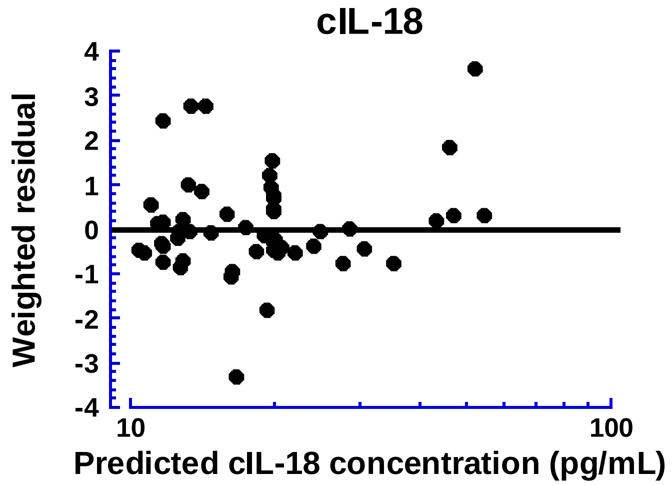

Supplementary Figure S5. Residual plots obtained from pharmacokinetic/pharmacodynamic modeling of average platelet count and hematocrit data in cynomolgus monkeys

A. Platelet count data

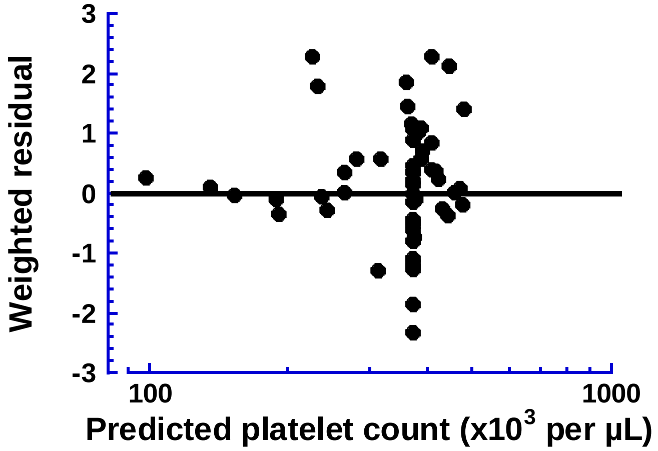

B. Hematocrit data

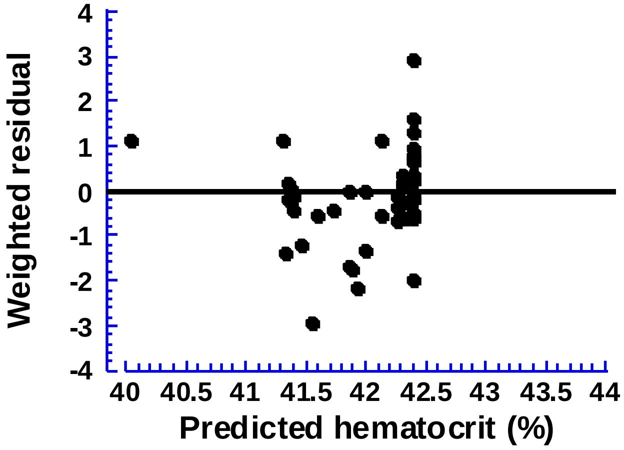

Supplementary Figure S6. Simulated cIL-18 induction by hFc-hIL-10 at the monkey efficacious dose of 0.025 mg/kg administered intravenously every 2, 3, or 4 weeks

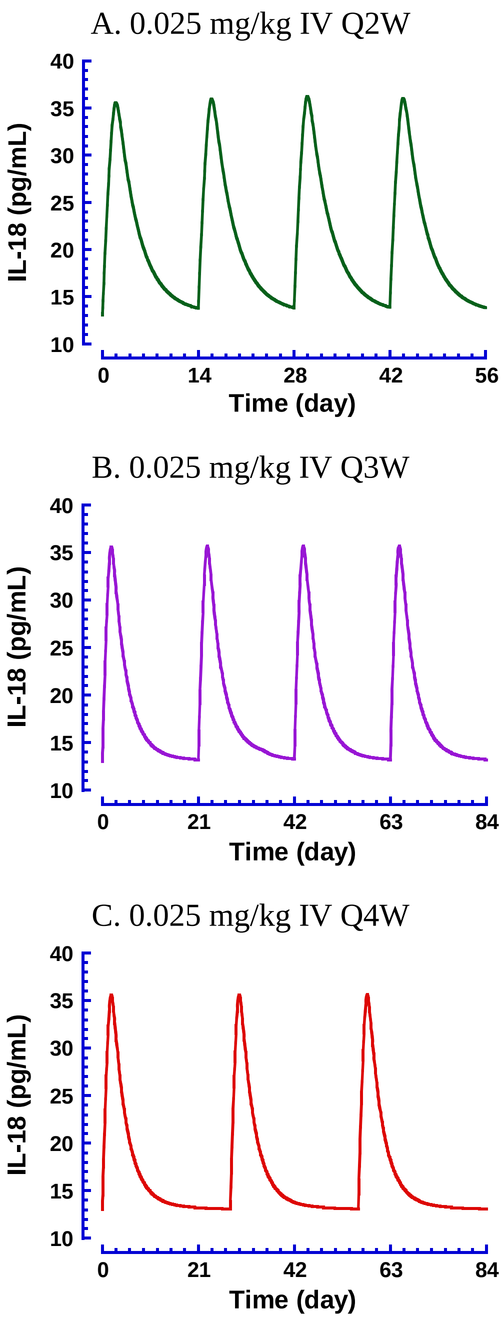

Supplement: Supplementary file 1 [file DataSheet1.pdf]
